# CD27 and ICOS as Targets of PD‐1/PD‐L1 Signaling to Regulate Resident Memory CD8^+^ T‐Cell‐Mediated Pulmonary Protection and Pathology

**DOI:** 10.1002/advs.202512452

**Published:** 2025-11-20

**Authors:** Yuanyuan Chen, Zhenzhen Wang, Liqiang Song, Qianqian Chen, Hang Zhang, Tao Pan, Jianli Pan, Sheng Zhao, Ting Shang, Hong'en Xu, Xing Lv, Jing Li, Fei Kang, Yongzhan Nie, Feng Zhao, Lei Shi, Zheng Wang

**Affiliations:** ^1^ Department of Pulmonary and Critical Care Medicine Xijing Hospital Fourth Military Medical University Xi'an 710032 China; ^2^ Cardiovascular Center Department of Ultrasound Medicine Affiliated People's Hospital Zhejiang Provincial People's Hospital Hangzhou Medical College Hangzhou 310014 China; ^3^ Department of Special Need Xi'an Children's Hospital Xi'an 710032 China; ^4^ State Key Laboratory of Holistic Integrative Management of Gastrointestinal Cancers National Clinical Research Center for Digestive Diseases Xijing Hospital of Digestive Diseases Fourth Military Medical University Xi'an 710032 China; ^5^ Cancer Center Department of Radiation Oncology Affiliated People's Hospital Zhejiang Provincial People's Hospital Hangzhou Medical College Hangzhou 310014 China; ^6^ Department of Nuclear Medicine Xijing Hospital Fourth Military Medical University Xi'an 710032 China

**Keywords:** costimulatory signaling, fibrotic sequelae, influenza, PD‐1/PD‐L1, resident memory T cells

## Abstract

Tissue‐resident memory CD8^+^ T cells (T_RM_ cells) provide superior frontline defense against pathogens. While the role of costimulation in effector and memory CD8^+^ T‐cell development is well characterized, how costimulatory signaling governs CD8^+^ T_RM_ cells homeostasis at the memory phase remains poorly defined. Here it is revealed that the costimulatory receptors CD27 and ICOS coordinately sustain PD‐1^high^ CD8^+^ T_RM_ cell populations following resolution of acute influenza infection. These costimulatory signals serve as critical targets for PD‐1/PD‐L1 blockade, thereby facilitating the rejuvenation of PD‐1^high^ T_RM_ cells and influencing the progression of fibrotic sequelae during the memory phase. Mechanistic dissection identifies the nuclear receptor Nur77 (NR4A1) as the convergent transcriptional hub downstream of CD27/ICOS, governing proliferative renewal and maintenance of PD‐1^high^ T_RM_ cells. Therapeutic administration of a CD27 agonist not only amplified this T_RM_ cell subset in late‐stage memory but also conferred cross‐protective immunity against heterosubtypic viral challenges. Clinically, the expressions of CD27 and ICOS are enriched in CD8^+^ T cells within the lung tissues of patients with pulmonary fibrosis. Collectively, these findings establish the “CD27/ICOS‐NR4A1‐proliferation” axis as a linchpin of PD‐1/PD‐L1‐mediated T_RM_ cell homeostasis, revealing druggable targets for intercepting infection‐associated fibrotic progression.

## Graphical abstract

Here it is revealed that the costimulatory receptors CD27 and ICOS are essential for the long‐term maintenance of PD‐1^high^ CD8^+^ T_RM_ cells following influenza infection. PD‐L1 blockade enhances the proliferation and activation of these cells through Nur77 activation downstream of the CD27/ICOS signaling axis, thereby boosting host immunity against secondary viral challenges while concurrently exacerbating pulmonary fibrosis sequelae.

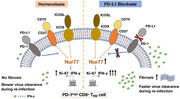

## 1 Introduction

Tissue‐resident memory CD8^+^ T cells (T_RM_ cells) stationed at mucosal barriers serve as first‐line defenders against pathogen reinfection through coordinated innate and adaptive immune activation.^[^ ^1^, ^2^, ^3^ ^]^ While substantial research efforts have focused on the differentiation dynamics and protective potentia of these sentinel cells,^[^ ^4^ ^]^ their dual‐edge nature in maintaining tissue homeostasis remains mechanistically obscure. Emerging evidence suggests that dysregulated T_RM_ responses may paradoxically drive chronic tissue damage,^[^ ^5^, ^6^, ^7^ ^]^ particularly in postviral pulmonary complications—a phenomenon demanding systematic investigation.

The global burden of influenza virus persists with >30 000 annual fatalities from pneumonia and fibrotic sequelae,^[^ ^8^, ^9^ ^]^ yet the immunological determinants of these pathological outcomes remain elusive. Following acute influenza infection in mice, our model reveals epitope‐specific dichotomy: NP_366–374_‐specific T_RM_ cells dominate secondary responses through clonal superiority, contrasting with PA_224–233_ counterparts in phenotypic signatures and functional programming.^[^ ^10^, ^11^, ^12^, ^13^, ^14^, ^15^ ^]^ Crucially, we previously identified PD‐1^high^ T_RM_ subpopulations exhibiting exhaustion‐like properties that require tight homeostatic control to prevent postclearance pulmonary fibrosis (PF).^[^ ^5^ ^]^ This finding positions PD‐1/PD‐L1 axis modulation as a critical regulator of T_RM_‐mediated pathogenesis, though the downstream effectors and costimulatory crosstalk remain uncharted territory.

Costimulatory molecules are constitutively expressed by naive T cells or induced upon T‐cell activation, playing an essential role in optimal clonal expansion and shaping subsequent memory differentiation of T cells.^[^ ^16^, ^17^ ^]^ One study demonstrated that NP_366–374_ and PA_224–233_ CD8^+^ T cells differentially require CD40 and CD27 for their memory programming during the primary response to influenza infection.^[^ ^10^ ^]^ A recent study reported that Inducible T‐cell costimulator (ICOS) expression in tissues is necessary for the establishment of CD8^+^ T_RM_ cells.^[^ ^18^ ^]^ While cell–cell interactions dictate the tissue residency and adaptation of memory CD8^+^ T cells, the role of costimulatory molecules in the maintenance of CD8^+^ T_RM_ cells—particularly in those mediating tissue pathogenesis—has not yet been explored.

The PD‐1/PD‐L1 axis serves as a critical immunoregulatory checkpoint that constrains CD8^+^ T‐cell activation to preserve peripheral tolerance.^[^ ^19^, ^20^, ^21^, ^22^ ^]^ Chronic antigen stimulation drives T‐cell exhaustion, manifesting as sustained PD‐1 overexpression in both tumor microenvironments and persistent viral infections.^[^ ^23^, ^24^ ^]^ Clinically, PD‐1/PD‐L1 blockade demonstrates therapeutic efficacy across multiple malignancies by reversing T‐cell exhaustion,^[^ ^25^ ^]^ though this intervention paradoxically triggers immune‐related adverse events (irAEs) in peripheral tissues.^[^ ^26^, ^27^, ^28^, ^29^ ^]^ PD‐1 is a hallmark of CD8^+^ T_RM_ cells, and we previously reported that PD‐1/PD‐L1 blockade at the memory stage following acute influenza infection results in the progression of severe fibrotic sequelae.^[^ ^5^ ^]^ This pathological conversion implies an underappreciated homeostatic role of PD‐1 signaling in curbing T_RM_‐mediated tissue damage. Furthermore, a major focus in this field is to clarify the mechanisms through which PD‐1/PD‐L1 blockade rejuvenates exhausted T cells. Recently, two studies demonstrated that CD28 is the primary target of PD‐1‐recruited SHP‐2, indicating that PD‐1 inhibits T‐cell function by inactivating CD28 signaling, with CD28/B7 costimulation being essential for the rescue of CD8^+^ T cells following PD‐1/PD‐L1 blockade.^[^ ^30^, ^31^ ^]^ Our previous work also confirmed that CD28/B7 is a target of PD‐1/PD‐L1 blockade for the rejuvenation of CD8^+^ T_RM_ cells, even in the context of acute viral infections.^[^ ^5^ ^]^ However, the involvement of alternative costimulatory axes in T_RM_‐mediated pathology remains unclear.

Here, we report that PD‐1^high^ CD8^+^ T_RM_ cells demonstrate continuous proliferation at the memory stage following influenza infection. CD27 and ICOS are highly expressed in these T_RM_ cells and are required for their persistent proliferation as well as long‐term maintenance. Furthermore, CD27 and ICOS have been shown to be targets of the PD‐1/PD‐L1 pathway, as anti‐CD70 or anti‐ICOS treatments abrogate the PD‐L1 blockade‐mediated reinvigoration of CD8^+^ T_RM_ cells and the associated pulmonary fibrotic sequelae. Mechanistically, the nuclear receptor Nur77 (NR4A1) is the key transcriptional factor downstream of CD27 and ICOS signaling and is responsible for the proliferative capacity and maintenance of PD‐1^high^ CD8^+^ T_RM_ cells. Based on these findings, the use of a CD27 agonist starting at the memory stage allows PD‐1^high^ CD8^+^ T_RM_ cells to be maintained for a much longer duration with no increase in tissue pathology, while providing better protection for secondary responses. Furthermore, CD27^+^ PD‐1^+^ and ICOS^+^ PD‐1^+^ CD8^+^ T cells were found in pulmonary fibrosis tissue compared to control samples. Our data imply that CD27 and ICOS are potential therapeutic targets for virus‐induced pulmonary fibrotic sequelae.

## 2 Results

### 2.1 Persistent Proliferation during the Memory Stage: A Hallmark of PD‐1^high^ CD8^+^ T_RM_ Cells, Which Gradually Lose This Capacity over Time

**Figure 1:**
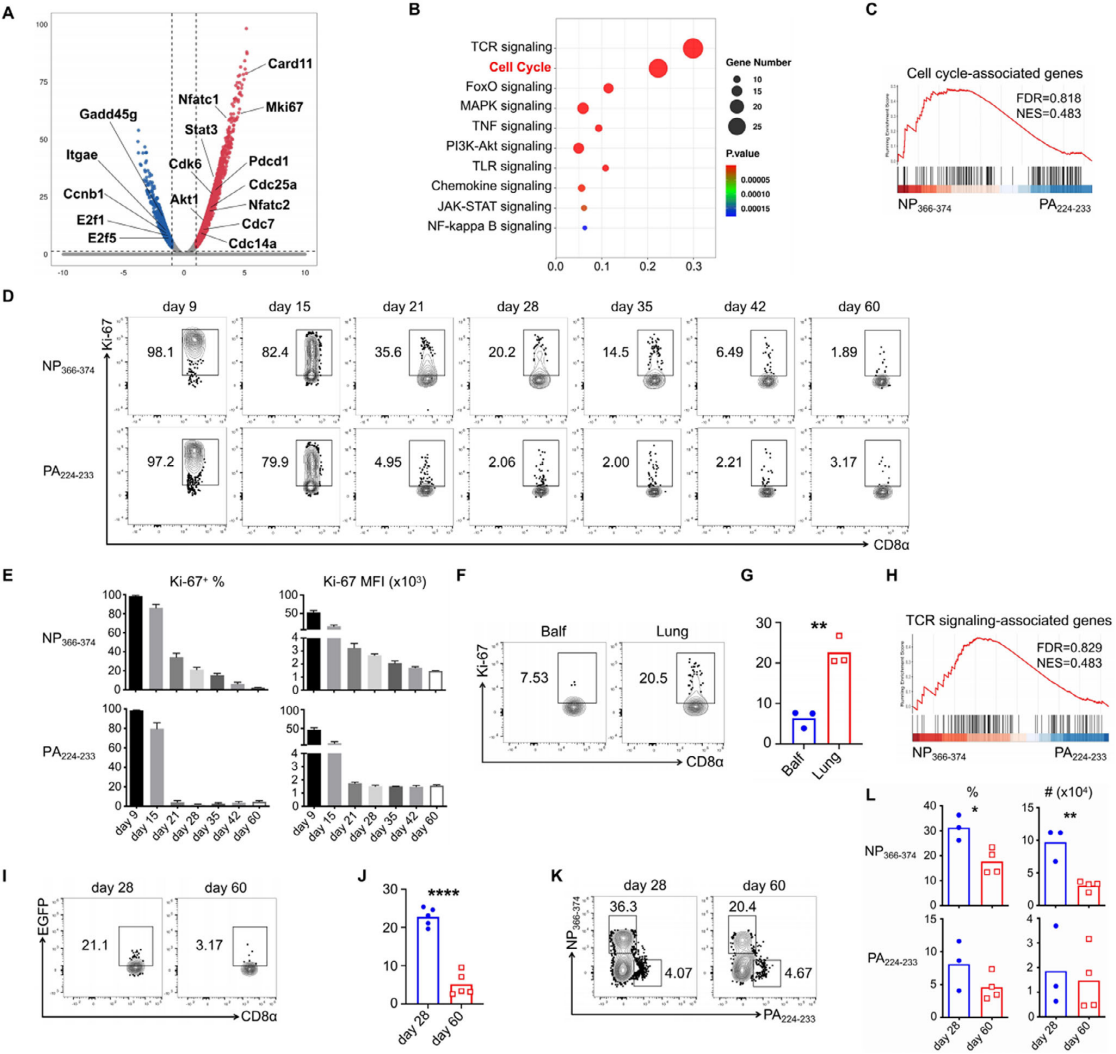
NP_366–374_ CD8^+^ T_RM_ cells exhibit enhanced persistent proliferative capacity in the lung during memory stage following acute influenza infection. A–C) WT C57BL/6 mice were infected with influenza PR8, NP_366–374_ and PA_224–233_ CD8^+^ T_RM_ cells were sorted for bulk RNA sequencing at 28 d.p.i. A) Volcano plot depicting differentially expressed genes between NP_366–374_ and PA_224–233_ T_RM_ cells. Red indicates upregulated genes in NP_366–374_ T_RM_ cells, while blue indicates those upregulated in PA_224–233_ T_RM_ cells. B) Gene Ontology (GO) pathway enrichment analysis comparing NP_366–374_ and PA_224–233_ T_RM_ cells. C) Gene Set Enrichment Analysis (GSEA) revealing positive enrichment of cell‐cycle‐associated genes in NP_366–374_ T_RM_ cells. D,E) WT C57BL/6 mice infected with influenza PR8 were sacrificed at specified d.p.i. D) Ki‐67 expression in lung NP_366–374_ and PA_224–233_ CD8^+^ T cells was evaluated by flow cytometry. E) The frequency and total cell numbers of Ki‐67^+^ NP_366–374_ and PA_224–233_ CD8^+^ T cells were quantified. F,G) WT C57BL/6 mice were infected with influenza PR8, and Ki‐67 expression of NP_366–374_ T_RM_ cells from bronchoalveolar lavage fluid (BALF) and lung tissue was assessed by flow cytometry at 28 d.p.i. H) GSEA illustrating positive enrichment of T‐cell receptor (TCR) signaling‐associated genes in NP_366–374_ T_RM_ cells. I,J) Nur77–EGFP transgenic mice infected with influenza PR8 were evaluated for EGFP expression in lung NP_366–374_ T_RM_ cells by flow cytometry at the designated d.p.i. K,L) WT C57BL/6 mice were rechallenged with influenza X31 at 28 or 60 d.p.i. in the presence of FTY720. Mice were sacrificed to assess the frequency and total cell numbers of NP_366–374_ and PA_224–233_ CD8^+^ T cells at day 4 post‐rechallenge. Representative of three to four experiments (*n* = 3–5) except panels (A–C and H). Data are mean ± standard deviation (SD). *p < 0.05, **p < 0.01, and ****p < 0.0001, unpaired two‐tailed *t*‐test.

Our previous research demonstrated epitope‐specific heterogeneity between NP_366–374_ and PA_224–233_ CD8^+^ T_RM_ cells following acute influenza virus infection.^[^ ^5^ ^]^ NP_366–374_ T_RM_ cells are the predominant cell type in lung tissue contributing to heterosubtypic immunity, primarily because all major histocompatibility complex class I (MHC‐I) positive cells can activate them, unlike other epitope‐specific counterparts.^[^ ^14^, ^32^ ^]^ Nevertheless, not all memory CD8^+^ T cells in the lung are long lasting. We and others have reported that CD8^+^ T_RM_ cells in the lung experience enhanced apoptosis and decline over time following influenza infection.^[^ ^5^, ^33^, ^34^ ^]^ Furthermore, our previous findings indicated that NP_366–374_ T_RM_ cells are lost more rapidly than their PA_224–233_ counterparts.^[^ ^5^ ^]^ However, the mechanisms underpinning the maintenance of these two epitope‐specific T_RM_ cells at the memory stage remain unclear. To address this question, we infected C57BL/6 mice with the influenza virus strain A/PR8/34 and sorted NP_366–374_ and PA_224–233_ T_RM_ cells from the lungs by a flow cytometer on day 28 post infection (Figure S1A, Supporting Information). We labeled the mice intravenously with a fluorescently coupled anti‐CD45 antibody (Ab) 5 min before tissue collection to differentiate between lung‐circulating (intravenous Ab^+^) and lung‐resident (intravenous Ab^−^) CD8^+^ T cells, as previously described.^[^ ^5^ ^]^ Subsequently, we performed bulk RNA sequencing (RNA‐seq) analysis on the sorted cells. Consistent with our earlier findings, NP_366–374_ T_RM_ cells exhibited elevated expression of *Pdcd1* (encoding PD‐1), while *Itgae* (encoding CD103) was expressed at higher levels in PA_224–233_ T_RM_ cells (**Figure** **1**A). Notably, NP_366–374_ T_RM_ cells displayed significantly higher levels of *Mki67* (encoding Ki‐67) and other cell‐cycle‐associated genes, indicating enhanced proliferative characteristics during the memory phase (Figure 1A). Pathway analysis and Gene Set Enrichment Analysis (GSEA) revealed that NP_366–374_ T_RM_ cells were positively enriched in genes associated with the cell cycle compared to PA_224–233_ T_RM_ cells (Figure 1B,C).

To further profile the kinetics of the proliferative capacities of NP_366–374_ and PA_224–233_ T_RM_ cells at the protein level, we assessed cell proliferation by intracellular staining for Ki‐67 at various time points post influenza infection. The Ki‐67 expression of NP_366–374_ and PA_224–233_ CD8^+^ T cells was comparable during both the effector (day 9) and contraction (day 15) phases (Figure 1D,E). Influenza virus infection typically resolves in infected lungs by ≈10 days post infection (d.p.i.), and our data align with this, as viral genes were undetectable after this time (Figure S1B, Supporting Information). However, NP_366–374_ T_RM_ cells maintained their proliferative capacity during the memory stage (day 21), whereas PA_224–233_ T_RM_ cells exhibited diminished proliferation starting at 21 d.p.i. (Figure 1D,E). Given that maintaining a balance between proliferation and apoptosis is crucial for sustaining the T‐cell pool within tissues, we next evaluated apoptosis in these CD8^+^ T cells at specified time points using Annexin V (AnnV) staining. No significant differences in cell death were noted between the two epitope‐specific T_RM_ cell types, as both demonstrated comparable levels of AnnV (Figure S1C, Supporting Information), suggesting that they receive similar signals for apoptosis. Furthermore, influenza infection resulted in T_RM_ cell retention in both the airways and the interstitium; thus, we compared Ki‐67 levels of NP_366–374_ T_RM_ cells at these distinct locations. Lung T_RM_ cells, as opposed to airway T_RM_ cells, exhibited persistent proliferation characteristics (Figure 1F,G). Overall, lung NP_366–374_ CD8^+^ T_RM_ cells display sustained proliferative capacity at the memory stage, a phenomenon previously undocumented in T_RM_ cells.

It is important to note that NP_366–374_ T_RM_ cells eventually lose their persistent proliferative capacity over time, evident by the scarcity of Ki‐67 positive cells at the late memory stage (day 60) (Figure 1D,E). We previously reported that NP_366–374_ T_RM_ cells experience prolonged T‐cell receptor (TCR)/CD28 signaling.^[^ ^5^ ^]^ Our GSEA data also indicated elevated expression of several TCR signaling‐associated genes in NP_366–374_ T_RM_ cells (Figure 1H). Using Nur77–Enhanced Green Fluorescent Protein (EGFP) transgenic mice to monitor TCR signaling activation, we examined Nur77–EGFP expression during early and late memory stages. Consistent with our Ki‐67 results, Nur77–EGFP levels in NP_366–374_ T_RM_ cells were lower at 60 d.p.i. compared to 21 d.p.i. (Figure 1I,J). Regarding the expression of PD‐1 on NP_366–374_ and PA_224–233_ T_RM_ cells, we observed that during the effector phase, both populations exhibited similar levels of PD‐1 expression (Figure S1D, Supporting Information). However, in the memory phase following infection, NP_366–374_ T_RM_ cells showed significantly higher PD‐1 expression compared to PA_224–233_ T_RM_ cells (Figure S1D, Supporting Information). Moreover, within the NP_366–374_ T_RM_ population, PD‐1 expression progressively declined over time (Figure S1D, Supporting Information). To further elucidate the relationship between PD‐1 expression, TCR signaling, and cell proliferation in NP_366–374_ T_RM_ cells, we stratified these cells according to PD‐1 expression levels and assessed Ki‐67 and Nur77–EGFP expressions. We observed a positive correlation between PD‐1 and Nur77–EGFP expressions, along with a negative correlation between PD‐1 and Ki‐67 (Figure S1E,F, Supporting Information), suggesting that NP_366–374_ T_RM_ cells are under persistent TCR stimulation, and that elevated PD‐1 expression in this subset is associated with suppressed proliferative activity.

Finally, we sought to determine whether the reduced proliferation observed in NP_366–374_ T_RM_ cells correlates with a diminished response to secondary reinfection. To investigate this, we infected C57BL/6 mice with the influenza strain PR8 and subsequently rechallenged them with the influenza X31 virus at either 28 or 60 d.p.i. We then evaluated T‐cell responses on day 4 post rechallenge; the frequency and cell numbers of day 60 NP_366–374_ T_RM_ cells were significantly decreased compared to day 40 NP_366–374_ T_RM_ cells, while the PA_224–233_ T_RM_ cells remained unchanged (Figure 1K,L). Collectively, these observations suggest that lung NP_366–374_ CD8^+^ T_RM_ cells receive TCR and/or costimulatory signals, allowing them to maintain persistent proliferative capacity during the early memory stage, although this capacity declines over time.

### 2.2 Negative Regulation of PD‐1^high^ CD8^+^ T_RM_ Cells by PD‐1, but Not Other Inhibitory Signaling Pathways

**Figure 2:**
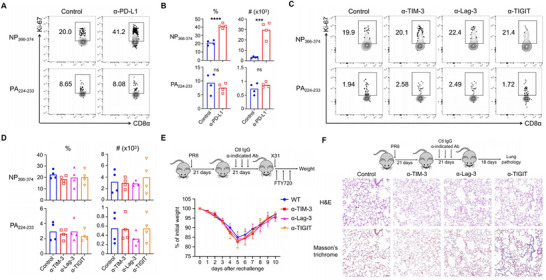
PD‐1/PD‐L1, not other co‐inhibitory pathways, negatively regulates the persistent proliferation and homeostasis of NP_366–374_ CD8^+^ T_RM_ cells. A,B) WT C57BL/6 mice were infected with influenza PR8 and received either control IgG or anti‐PD‐L1 (α‐PD‐L1) from 21 to 25 d.p.i. Lung was harvested following intravenous administration of CD45 antibodies. A) Representative plots of Ki‐67^+^ NP_366–374_ and PA_224–233_ T_RM_ cells at 29 d.p.i. B) Frequencies and total cell numbers of Ki‐67^+^ NP_366–374_ and PA_224–233_ T_RM_ cells at 29 d.p.i. C,D) WT C57BL/6 mice were infected with influenza PR8 and received control IgG, anti‐TIM‐3 (α‐TIM‐3), anti‐Lag‐3 (α‐Lag‐3), or anti‐TIGIT (α‐TIGIT) treatment from 21 to 25 d.p.i. C) Representative plots of Ki‐67^+^ NP_366–374_ and PA_224–233_ T_RM_ cells at 29 d.p.i. D) Frequencies and total cell numbers of Ki‐67^+^ NP_366–374_ and PA_224–233_ T_RM_ cells at 29 d.p.i. E) WT C57BL/6 mice were infected with influenza PR8 and received control IgG, α‐TIM‐3, α‐Lag‐3 or α‐TIGIT from 21 to 37 d.p.i., followed by rechallenge with X31 (2 × 10^4^ pfu) at 42 d.p.i. in the presence of FTY720. Schematic of experimental design (top) and percentages of original body weight following rechallenge were assessed daily. F) WT C57BL/6 mice were infected with influenza PR8 received control IgG, α‐TIM‐3, α‐Lag‐3, or α‐TIGIT from 21 to 37 d.p.i. Lung pathology was evaluated at 60 d.p.i. Representative of three experiments (*n* = 4). Data are mean ± SD; ns, not significant. ***p < 0.001, **** *p* < 0.0001, unpaired two‐tailed *t*‐test.

Virus‐specific CD8^+^ T cells that provide the proliferative burst after anti‐PD‐1/PD‐L1 therapy in mice chronically infected with lymphocytic choriomeningitis virus (LCMV).^[^ ^35^ ^]^ Our previous study reported that PD‐1/PD‐L1 blockade promotes reinvigoration of NP_366–374_ T_RM_ cells.^[^ ^5^ ^]^ This led us to investigate whether anti‐PD‐1/PD‐L1 therapy also enhances the proliferation of these cells during acute influenza infection. We infected C57BL/6 mice with influenza PR8 and treated them with anti‐PD‐L1 (α‐PD‐L1) from 21 to 41 d.p.i. At 45 d.p.i., we evaluated T_RM_ cell proliferation by examining Ki‐67 expression. Unexpectedly, while PD‐L1 blockade reduced the Ki‐67 expression of NP_366–374_ T_RM_ cells, both their frequency and total cell numbers increased (Figure S2A–D, Supporting Information). Given that we assessed T_RM_ cell proliferation after five treatments with α‐PD‐L1, it is plausible that these cells had already given rise to daughter cells through a proliferative burst. Therefore, we modified our experiment to start at 21 d.p.i. and examined Ki‐67 at 29 d.p.i. after two treatments with α‐PD‐L1. The blockade of PD‐1/PD‐L1 interaction significantly increased the percentages and total cell numbers of Ki‐67^+^ NP_366–374_ T_RM_ cells, whereas no such effect was observed for Ki‐67^+^ PA_224–233_ T_RM_ cells (**Figure** **2**A,B).

In chronic viral infections and cancer, a hallmark of exhausted CD8^+^ T cells is the expression of various inhibitory receptors. In addition to PD‐1, we previously reported that lung NP_366–374_ T_RM_ cells express higher levels of TIM‐3, Lag‐3, and TIGIT compared to PA_224–233_ T_RM_ cells;^[^ ^5^ ^]^ however, their function in regulating T_RM_ cells remains unclear. Consequently, we sought to examine the role of these other inhibitory receptors in the proliferation of NP_366–374_ T_RM_ cells. We infected C57BL/6 mice with influenza PR8 and treated them separately with anti‐TIM‐3 (α‐TIM‐3), anti‐Lag‐3 (α‐Lag‐3), and anti‐TIGIT (α‐TIGIT) from days 21 to 25 d.p.i., assessing Ki‐67 levels at 29 d.p.i. However, the blockade of any of these inhibitory receptors did not affect the proliferation of NP_366–374_ T_RM_ cells (Figure 2C,D). Consistent with this finding, mice treated with these antibodies from days 21 to 41 d.p.i. showed no change in the frequency or total cell numbers of NP_366–374_ T_RM_ cells at 45 d.p.i. (Figure S2E,F, Supporting Information). Furthermore, blocking TIM‐3, Lag‐3, and TIGIT did not provide PR8‐infected mice with improved protection against heterologous reinfection (Figure 2E) and did not affect lung inflammation or fibrotic sequelae (Figure 2F; Figure S2G, Supporting Information), contrasting sharply with the effects of α‐PD‐L1 at the T_RM_ stage.

Combination therapy targeting multiple checkpoints can significantly enhance CD8^+^ T‐cell responses compared to monoclonal antibody blockade alone. Therefore, we combined α‐TIM‐3, α‐Lag‐3, and α‐TIGIT and treated PR8‐infected C57BL/6 mice starting at 21 d.p.i. Similar to the effects observed with single antibody blockade, the combination blockade did not influence proliferation, frequency, total cell numbers, heterologous immunity, or lung histology (Figure S2H–N, Supporting Information). These data suggest that only PD‐1/PD‐L1 signaling inhibits the proliferation and maintenance of NP_366–374_ T_RM_ cells during the memory stage following influenza infection, while other inhibitory pathways do not play a significant role.

### 2.3 The Necessity of Costimulatory Receptors CD27 and ICOS for the Long‐Term Maintenance of PD‐1^high^ CD8^+^ T_RM_ Cells

A notable characteristic of CD8^+^ T_RM_ is the upregulation of costimulatory receptor genes, particularly in comparison to their circulating memory counterparts.^[^ ^36^ ^]^ Costimulatory signaling is essential for the expansion of naive T cells and their differentiation into memory cells.^[^ ^37^ ^]^ In our previous study, we reported that the interaction between CD28 and B7 is critical for the long‐term maintenance of NP_366–374_ T_RM_ cells.^[^ ^5^ ^]^ This raises the question of whether other costimulatory molecules play similar roles in supporting the maintenance of NP_366–374_ T_RM_ cells.

**Figure 3:**
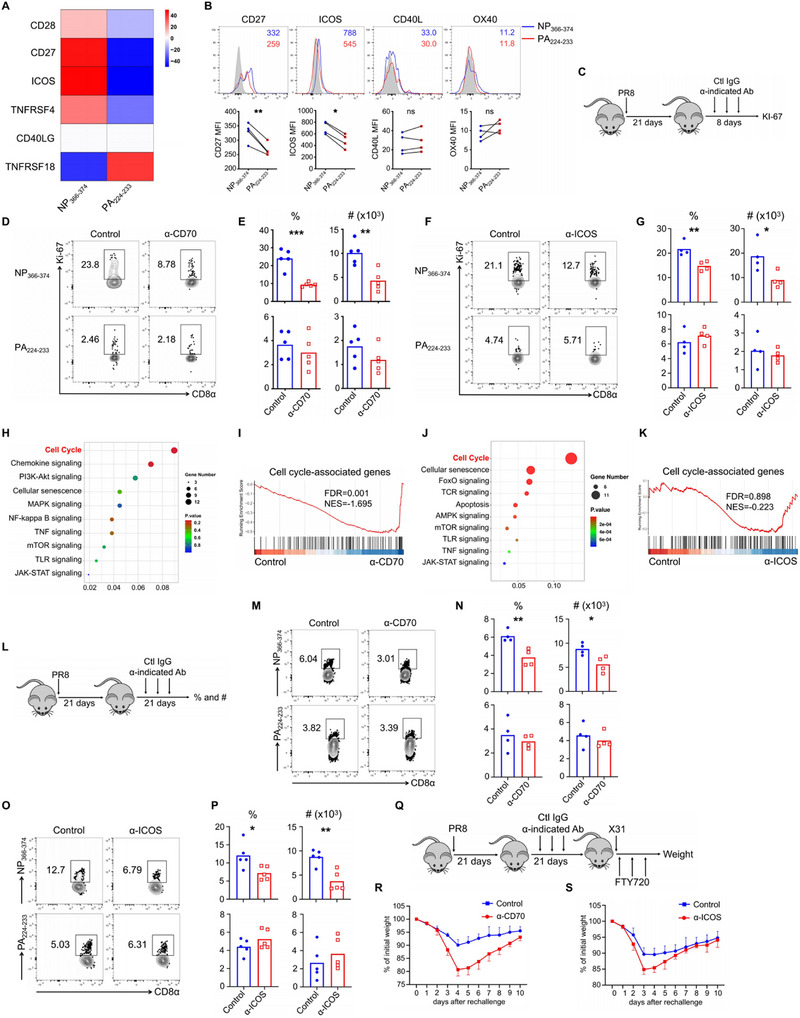
CD27–CD70 and ICOS–ICOSL interactions are required for the long‐term maintenance of NP_366–374_ CD8^+^ T_RM_ cells. A) Heatmap depicting the differential expression of various costimulatory receptors in NP_366–374_ versus PA_224–233_ T_RM_ cells at 28 d.p.i., based on original data from Figure 1A–D. B) Flow cytometry was used to confirm the differential expression of CD27, ICOS, CD40L, and OX40 on NP_366–374_ or PA_224–233_ T_RM_ cells through surface staining for CD27, CD40L, and OX40, and intracellular staining for ICOS at 28 d.p.i. C–G) WT C57BL/6 mice were infected with influenza PR8 and received either control IgG, anti‐CD70 (α‐CD70), or anti‐ICOS (α‐ICOS) from 21 to 25 d.p.i. Lung was harvested following intravenous administration of CD45 antibodies. C) Schematic of experimental design. D,F) Representative plots of Ki‐67^+^ NP_366–374_ and PA_224–233_ T_RM_ cells at 29 d.p.i. E,G) Frequencies and total cell numbers of Ki‐67^+^ NP_366–374_ and PA_224–233_ T_RM_ cells at 29 d.p.i. H) Gene Ontology (GO) pathway enrichment analysis of NP_366–374_ T_RM_ cells comparing control and α‐CD70 groups. I) Gene Set Enrichment Analysis (GSEA) showing negative enrichment of cell‐cycle‐associated genes in NP_366–374_ T_RM_ cells from the α‐CD70 group. J) Go pathway enrichment analysis of NP_366–374_ T_RM_ cells comparing control and α‐ICOS groups. K) GSEA showing negative enrichment of cell‐cycle‐associated genes in NP_366–374_ T_RM_ cells from the α‐ICOS group. L–P) WT C57BL/6 mice were infected with influenza PR8 and received either control IgG, α‐CD70, or α‐ICOS from 21 to 37 d.p.i. L) Schematic of experimental design. M,O) Representative plots of Ki‐67^+^ NP_366–374_ and PA_224–233_ T_RM_ cells at 42 d.p.i. N,P) Frequencies and total cell numbers of Ki‐67^+^ NP_366–374_ and PA_224–233_ T_RM_ cells at 42 d.p.i. Q,S) WT C57BL/6 mice were infected with influenza PR8 and received control IgG, R) α‐CD70 or S) α‐ICOS from 21 to 37 d.p.i., followed by rechallenge with X31 (1×10^4^ pfu) at 42 d.p.i. in the presence of FTY720. Percentages of original body weight following rechallenge were assessed daily. Q) Schematic of experimental design. Representative of three to four experiments (*n* = 4–5) except panels (A and H–K). Data are mean ± SD. *p < 0.05, ** *p* < 0.01, and *** *p* < 0.001, unpaired two‐tailed *t*‐test.

Utilizing the bulk RNA sequencing data presented in Figure 1, we first compared the expression levels of costimulatory receptors between NP_366–374_ and PA_224–233_ T_RM_ cells. Our analysis revealed that several costimulatory receptors—including CD28, CD27, ICOS, and OX40—were expressed at higher levels in NP_366–374_ T_RM_ cells compared to PA_224–233_ T_RM_ cells (**Figure** **3**A). We subsequently validated these findings at the protein level at 28 d.p.i. In contrast to the RNA sequencing data, we observed that CD27 and ICOS were expressed at higher levels in NP_366–374_ T_RM_ cells, while OX40 and CD40L levels were comparable between the two T_RM_ cell types (Figure 3B).

We conducted a series of independent experiments to further examine the influence of these costimulatory receptors on the proliferation of NP_366–374_ T_RM_ cells. C57BL/6 mice were infected with the influenza PR8 and subsequently treated with control antibodies, anti‐CD70 (α‐CD70), anti‐ICOS (α‐ICOS), anti‐OX40 (α‐OX40), or anti‐CD40L (α‐CD40L) beginning at 21 d.p.i. Following two rounds of treatment by day 29, blockade of CD27–CD70 or ICOS–ICOSL resulted in a reduced frequency and number of Ki‐67^+^ NP_366–374_ T_RM_ cells, while not affecting Ki‐67^+^ PA_224–233_ T_RM_ cells (Figure 3C–G). Conversely, blockade of CD40–CD40L or OX40–OX40L did not influence the proliferation of either NP_366–374_ or PA_224–233_ T_RM_ cells (Figure S3A–D, Supporting Information). Thus, the sustained proliferation of NP_366–374_ T_RM_ cells is maintained through CD27–CD70 and ICOS–ICOSL signaling during the early memory stage following acute influenza infection. It is important to note that CD27 and/or ICOS are also expressed by other immune cells in addition to CD8^+^ T cells, particularly CD4^+^ T cells. For instance, ICOS is crucial for the function of regulatory T cells (Tregs). Therefore, it is possible that the role of α‐CD70 or α‐ICOS in reducing cell proliferation in NP_366–374_ T_RM_ cells may involve effects on other cell types. To investigate this possibility, we depleted CD4^+^ T cells starting at 21 d.p.i. and subsequently examined Ki‐67 expression at 29 d.p.i. Our data indicated that CD4 depletion did not affect the Ki‐67 expression of NP_366–374_ T_RM_ cells (Figure S3E,F, Supporting Information). To further investigate whether NP_366–374_ T_RM_ cell proliferation remains regulated by CD27/CD70 and ICOS/ICOSL signaling following CD4^+^ T‐cell depletion, we assessed the effects of α‐CD70 or α‐ICOS treatment on Ki‐67 expression in these cells. We found that blockade with α‐CD70 or α‐ICOS still reduced Ki‐67 expression in NP_366–374_ T_RM_ cells, indicating that these costimulatory pathways continue to modulate T_RM_ cell proliferation even in the absence of CD4^+^ T cells (Figure S3G–J, Supporting Information).

To gain further insight into the regulatory role of CD27–CD70 or ICOS–ICOSL interactions on the persistent proliferation of NP_366–374_ T_RM_ cells, we sorted these cells and performed bulk RNA sequencing after two rounds of treatment with α‐CD70 or α‐ICOS during the memory stage following influenza PR8 infection. The RNA sequencing analysis revealed that several signaling pathways, including those associated with the cell cycle, were significantly affected by α‐CD70 treatment at the memory stage (Figure 3H). GSEA confirmed that cell‐cycle‐associated genes were downregulated in the α‐CD70 treatment group (Figure 3I). Similarly, α‐ICOS treatment was associated with a trend toward downregulation of cell‐cycle‐related genes in NP_366–374_ T_RM_ cells (Figure 3J,K). To further investigate the relationship between CD27 or ICOS expression and cell proliferation, we sorted cells based on high versus low expression of CD27 or ICOS and performed bulk RNA sequencing at 28 d.p.i. RNA sequencing analysis revealed that cell‐cycle‐associated genes were significantly correlated with CD27^high^ or ICOS^high^ NP_366–374_ T_RM_ cells (Figure S4A–E, Supporting Information). Similarly, CD27^high^ NP_366–374_ T_RM_ cells displayed elevated levels of Nur77–EGFP and Ki‐67, as well as increased production of IFN‐γ (Interferon gamma) (Figure S4F–H, Supporting Information). The expression of PD‐1 on NP_366–374_ T_RM_ cells is also proportional to CD27 (Figure S4I, Supporting Information). Additionally, comparable results were observed when analyzing ICOS^high^ versus ICOS^low^ NP_366–374_ T_RM_ cells (Figure S4J–M, Supporting Information). The above results suggest a close correlation between CD27 or ICOS signaling and the proliferative capacity of NP_366–374_ T_RM_ cells during the early memory stage.

We further investigated the role of CD27–CD70 or ICOS–ICOSL interactions on modulating CD8^+^ T‐cell responses following influenza infection. Neither the α‐CD70 nor α‐ICOS blockade impacted the weight loss of mice during the early stages following acute PR8 infection (Figures S5A and S6A, Supporting Information). However, α‐CD70 treatment significantly impaired the generation of NP_366–374_ and PA_224–233_ epitope‐specific effector CD8^+^ T cells in the lung and spleen (Figure S5B–E, Supporting Information), while α‐ICOS did not affect the primary response to influenza infection (Figure S6B–E, Supporting Information). We subsequently assessed whether these costimulatory signals influence the responses of CD8^+^ T_RM_ cells. C57BL/6 mice infected with influenza PR8 were treated with α‐CD70 or α‐ICOS from 21 to 41 d.p.i., and T_RM_ cell responses were evaluated at 45 d.p.i. Both α‐CD70 and α‐ICOS treatments resulted in decreased frequency and cell numbers of NP_366–374_ T_RM_ cells but did not affect PA_224–233_ T_RM_ cells (Figure 3L–P). In addition, we found that treatment with α‐CD70 or α‐ICOS impaired the capacity of NP_366–374_ T_RM_ cells to produce IFN‐γ, demonstrating that CD27/CD70 and ICOS/ICOSL signaling pathways are essential for the maintenance of their effector functions (Figure S4N,O, Supporting Information). To determine whether impaired maintenance of NP_366–374_ T_RM_ cells decreases host resistance to heterologous reinfection, we infected and treated mice with α‐CD70 or α‐ICOS as described above. All mice were intraperitoneally injected with FTY720 and subsequently rechallenged with the influenza X31 virus at 45 d.p.i. Mice treated with either α‐CD70 or α‐ICOS exhibited greater weight loss compared to control mice (Figure 3Q–S). Collectively, these findings suggest that persistent CD27 and ICOS signaling are essential for the long‐term maintenance of NP_366–374_ CD8^+^ T_RM_ cells and are indispensable for providing protection against heterologous immunity.

### 2.4 CD27 and ICOS as Targets for PD‐1/PD‐L1‐Mediated Inhibition in PD‐1^high^ CD8^+^ T_RM_ Cells

**Figure 4:**
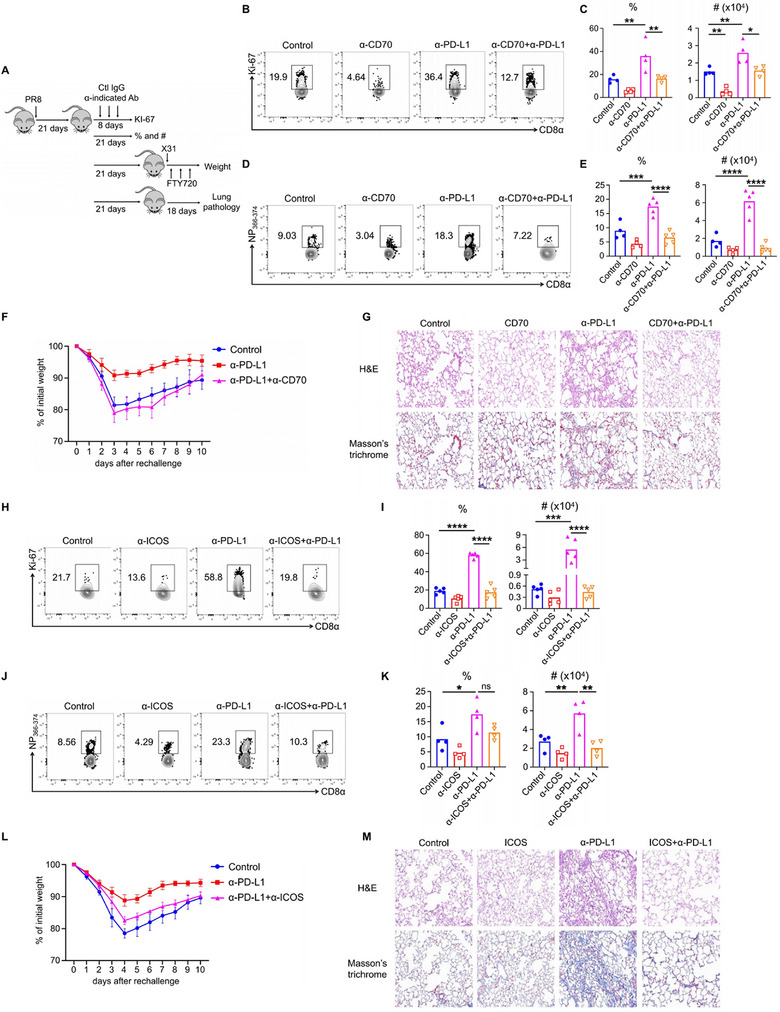
CD27–CD70 and ICOS–ICOSL as targets of α‐PD‐1/PD‐L1‐mediated rejuvenation of NP_366–374_ CD8^+^ T_RM_ cells. A–G) WT C57BL/6 mice were infected with influenza PR8 and received either control IgG, α‐PD‐L1, or α‐PD‐L1 plus α‐CD70 from 21 to 25 d.p.i., and continued until 37 d.p.i. A) Schematic of experimental design. B,C) Representative plots, frequency, and total cell numbers of Ki‐67^+^ NP_366–374_ T_RM_ cells at 29 d.p.i. D,E) Representative plots, frequencies and total cell numbers of NP_366–374_ T_RM_ cells at 42 d.p.i. F) All mice were rechallenged with X31 (2×10^4^ pfu) at 42 d.p.i. in the presence of FTY720. Percentages of original body weight following rechallenge were assessed daily. G) Lung pathology was evaluated at 60 d.p.i. H–M) Similar experiments similar to panels (B–G) were conducted, with α‐CD70 replaced by α‐ICOS. Representative of three experiments (*n* = 4–5). Data are mean ± SD; ns, not significant. * *p* < 0.05, ** *p* < 0.01, *** *p* < 0.001, and **** *p* < 0.0001, one‐way analysis of variance (ANOVA) with Tukey multiple comparison test.

The blockade of PD‐1 and PD‐L1 interactions promotes the rejuvenation of exhausted CD8^+^ T cells. Our previous study demonstrated that PD‐1 signaling inhibits CD28 function in NP_366–374_ T_RM_ cells during acute influenza infection.^[^ ^5^ ^]^ In addition to CD28, we further explored the roles of CD27 and ICOS in the response to PD‐1/PD‐L1 blockade. To investigate this, we infected C57BL/6 mice with influenza PR8 and subsequently administered control antibody, α‐CD70, α‐PD‐L1, or a combination of α‐CD70 and α‐PD‐L1 starting at 21 d.p.i. The coblockade of CD27 and PD‐1 signaling abrogated the effects of α‐PD‐L1 on the proliferative burst of NP_366–374_ T_RM_ cells at 29 d.p.i. (**Figure** **4**B,C) and their maintenance at 45 d.p.i. (Figure 4D,E). Moreover, coblockade impaired the α‐PD‐L1‐mediated enhancement of protective immunity against heterologous reinfection (Figure 4F). As previously reported, α‐PD‐L1 induced lung inflammation and persistent fibrotic sequelae after the resolution of acute influenza infection, while α‐CD70 counteracted the effects of α‐PD‐L1 on pulmonary fibrotic sequelae (Figure 4F; Figure S7A, Supporting Information).

Subsequently, we performed similar experiments by administering α‐ICOS to mice to investigate the role of ICOS and PD‐L1 coblockade in the response of NP_366–374_ T_RM_ cells. Like α‐CD70, the combination of α‐ICOS with α‐PD‐L1 reduced the proliferative burst and maintenance of NP_366–374_ T_RM_ cells (Figure 4H–K), impaired host defense against heterologous immunity (Figure 4L), and alleviated persistent fibrotic sequelae (Figure 4M; Figure S7B, Supporting Information). Collectively, the rejuvenation of NP_366–374_ T_RM_ cells by α‐PD‐L1 during the memory phase is dependent on both CD27 and ICOS signaling.

### 2.5 The Intrinsic Role of Costimulatory Signaling through NR4A1 in Regulating the Homeostasis of PD‐1^high^ CD8^+^ T_RM_ Cells

We next investigated the underlying mechanism by which transcriptional factors downstream of CD27 and ICOS signaling regulate NP_366–374_ T_RM_ cells. Several transcriptional factors have been reported to play critical roles in the differentiation and establishment of T_RM_ cells.^[^ ^36^, ^38^, ^39^ ^]^ Previous studies have indicated that NR4A1 is essential for the formation of exhausted T cells, and targeting it could enhance cancer immunotherapy.^[^ ^40^, ^41^ ^]^ Our prior research also established that NR4A1 is necessary for the generation of NP_366–374_ T_RM_ cells following influenza infection, with CD28/B7 blockade impairing NR4A1 expression in these cells.^[^ ^5^ ^]^

**Figure 5:**
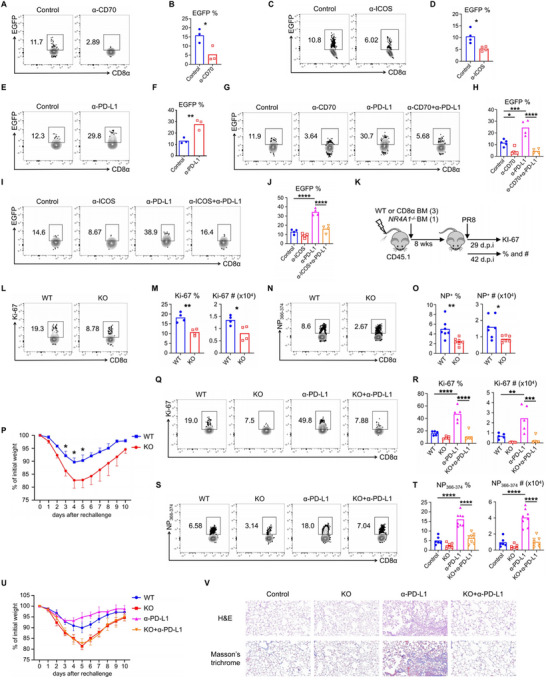
NR4A1 plays an intrinsic role in the regulation of NP_366–374_ CD8^+^ T_RM_ cells. A–J) Nur77–EGFP mice were infected with influenza PR8 and received either control IgG or the indicated antibodies from 21 to 25 d.p.i. Representative plots and frequencies of EGFP^+^ NP_366–374_ T_RM_ cells are shown at 29 d.p.i. K–O) Bone marrow chimeric mice were infected with influenza PR8. K) Schematic of experimental design. L,M) Representative plots, frequency, and total cell numbers of Ki‐67^+^ NP_366–374_ T_RM_ cells at 28 d.p.i. N,O) Representative plots, frequencies, and total cell numbers of NP_366–374_ T_RM_ cells at 42 d.p.i. P) WT and NR4A1^−/−^ mice were infected with influenza PR8 and rechallenged with X31 (1×10^4^ pfu) at 42 d.p.i. in the presence of FTY720. Percentages of original body weight following rechallenge were assessed daily. Q–V) WT and NR4A1^−/−^ mice were infected with influenza PR8 and received control IgG or α‐PD‐L1 from 21 to 25 d.p.i. or from 21 to 37 d.p.i. Q,R) Representative plots, frequency and total cell numbers of Ki‐67^+^ NP_366–374_ T_RM_ cells at 29 d.p.i. S,T) Representative plots, frequencies and total cell numbers of NP_366–374_ T_RM_ cells at 42 d.p.i. U) All mice were rechallenged with X31 (1×10^4^ pfu) at 42 d.p.i. in the presence of FTY720. Percentages of original body weight following rechallenge were assessed daily. V) Lung pathology was evaluated at 60 d.p.i. Representative of three to four experiments (*n* = 3–8). Data are mean ± SD. * *p* < 0.05, ** *p* < 0.01, *** *p* < 0.001, and **** *p* < 0.0001, unpaired two‐tailed *t*‐test or one‐way analysis of variance (ANOVA) with Tukey multiple comparison test.

To determine whether CD27 and ICOS control NR4A1 (encoding Nur77) expression in NP_366–374_ T_RM_ cells, we infected Nur77–EGFP mice with influenza PR8 and treated the mice with α‐CD70 or α‐ICOS starting at 21 d.p.i. We assessed EGFP levels in NP_366–374_ T_RM_ cells after the two treatments at 29 d.p.i. Our findings indicated that blockade of both CD70 and ICOS decreased the frequencies of EGFP^+^ NP_366–374_ T_RM_ cells (**Figure** **5**A–D), suggesting that persistent Nur77 expression in these cells at the memory stage following acute influenza infection is under the regulation of CD27 and ICOS signaling. We further examined the relationship between α‐PD‐L1 and Nur77 expressions, finding that α‐PD‐L1 increased EGFP levels at 29 d.p.i. (Figure 5E,F). Importantly, the increase in EGFP levels induced by α‐PD‐L1 was abrogated by α‐CD70 (Figure 5G,H) or α‐ICOS (Figure 5I,J) in NP_366–374_ T_RM_ cells, indicating that NR4A1 expression is tightly regulated by costimulatory and coinhibitory signals during the memory phase following influenza infection.

We previously reported that NR4A1 deficiency impairs the generation of NP_366–374_ T_RM_ cells;^[^ ^5^ ^]^ however, it remains unclear whether the persistent proliferation of these cells is controlled by NR4A1. To elucidate the role of NR4A1 in the proliferative capacity of NP_366–374_ T_RM_ cells, we infected wild‐type (WT) and NR4A1^−/−^ mice with influenza PR8. We observed that NR4A1 deficiency decreased the frequency and cell numbers of Ki‐67^+^ NP_366–374_ T_RM_ cells (Figure S8A,B, Supporting Information). Consistent with our earlier findings, the absence of NR4A1 impaired the maintenance of NP_366–374_ T_RM_ cells but not that of PA_224–233_ T_RM_ cells (Figure S8C,D, Supporting Information). NR4A1 deficiency also resulted in a decrease in PD‐1 expression on NP_366–374_ T_RM_ cells, while exhibiting no effect on PA_224–233_ T_RM_ cells (Figure S8E, Supporting Information). Furthermore, the absence of NR4A1 impairs the capacity of NP_366–374_ T_RM_ cells to produce IFN‐γ (Figure S8F, Supporting Information). In contrast, NR4A1 deficiency did not influence effector CD8^+^ T‐cell responses in the lung and spleen at 10 d.p.i. (Figure S9A–F, Supporting Information), suggesting that NR4A1 specifically participates in memory‐phase CD8^+^ T‐cell responses following acute influenza infection. To confirm that Nur77 plays an intrinsic role in the control of proliferation and generation of NP_366–374_ T_RM_ cells, we created CD8α knockout and NR4A1^−/−^ bone marrow chimeric mice to specifically deplete NR4A1 in CD8^+^ T cells. Similarly, NR4A1 deficiency in CD8^+^ T cells led to decreased frequency and cell numbers of Ki‐67^+^ NP_366–374_ T_RM_ cells at 28 d.p.i. (Figure 5K–M) as well as NP_366–374_ T_RM_ cells at 42 d.p.i. (Figure 5N,O). Furthermore, NR4A1 deficiency in CD8^+^ T cells impaired the host defense against heterologous reinfection (Figure 5P).

Given that α‐PD‐L1 rejuvenates NP_366–374_ T_RM_ cells at the memory stage, we investigated the role of NR4A1 deficiency in α‐PD‐L1‐mediated proliferative bursts in these cells. We infected WT and NR4A1 knockout mice with influenza PR8 and treated them with α‐PD‐L1 twice from 21 to 25 d.p.i. We subsequently examined the proliferation of NP_366–374_ T_RM_ cells at 28 d.p.i. PD‐L1 blockade increased both the frequency and cell numbers of Ki‐67^+^ NP_366–374_ T_RM_ cells; however, this effect was abrogated in NR4A1 knockout mice (Figure 5Q,R). We also treated the infected mice with α‐PD‐L1 from 21 to 41 d.p.i. and assessed T_RM_ cell responses at 42 d.p.i. NR4A1 deficiency abrogated the α‐PD‐L1‐mediated increases in frequency and number of NP_366–374_ T_RM_ cells (Figure 5S,T). Consistent with these findings, NR4A1 deficiency impaired the α‐PD‐L1‐mediated enhancement of heterologous protection (Figure 5U), alleviating lung inflammation and the development of fibrotic sequelae (Figure 5V; Figure S10, Supporting Information). Collectively, these data indicate that NR4A1, downstream of CD27 and ICOS signaling, is crucial for maintaining NP_366–374_ CD8^+^ T_RM_ cells and for responding to α‐PD‐L1 during the memory phase following acute influenza infection.

### 2.6 Enhanced CD27 Signaling in PD‐1^high^ CD8^+^ T_RM_ Cells Improves Long‐Term Heterologous Immunity in Hosts

**Figure 6:**
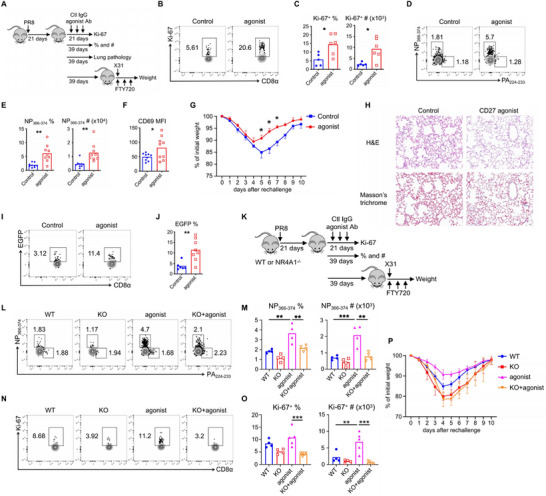
CD27 agonist provides better protection to hosts by maintaining NP_366–374_ CD8^+^ T_RM_ cells. A–H) WT C57BL/6 mice were infected with influenza PR8 and treated with either control IgG or CD27 agonist from 21 to 37 d.p.i. or from 21 to 57 d.p.i. A) Schematic of experimental design. B,C) Representative plots, frequencies, and total cell numbers of Ki‐67^+^ NP_366–374_ T_RM_ cells at 42 d.p.i. D,E) Representative plots, frequencies, and total cell numbers of NP_366–374_ T_RM_ cells at 60 d.p.i. F) CD69 expression level [mean florescence intensity (MFI)] on lung NP_366–374_ T_RM_ cells at 60 d.p.i. G) All mice were rechallenged with X31 (2×10^4^ pfu) at 60 d.p.i. in the presence of FTY720. Percentages of original body weight after rechallenge were assessed daily. H) Lung pathology was evaluated at 60 d.p.i. I,J) Nur77–EGFP mice were infected with influenza PR8 and received either control IgG or CD27 agonist from 21 to 37 d.p.i. Representative plots and frequencies of EGFP^+^ NP_366–374_ T_RM_ cells at 42 d.p.i. are shown. K–P) WT and NR4A1^−/−^ mice were infected with influenza PR8 and treated with either control IgG or CD27 agonist from 21 to 37 d.p.i. or from 21 to 57 d.p.i. K) Schematic of experimental design. L,M) Representative plots, frequencies and total cell numbers of NP_366–374_ T_RM_ cells at 60 d.p.i. N,O) Representative plots, frequencies, and total cell numbers of Ki‐67^+^ NP_366–374_ T_RM_ cells at 42 d.p.i. P) All mice were rechallenged with X31 (2×10^4^ pfu) at 60 d.p.i. in the presence of FTY720. Percentages of original body weight following rechallenge were assessed daily. Representative of three to four experiments (*n* = 4–9). Data are mean ± SD. * *p* < 0.05, ** *p* < 0.01, and *** *p* < 0.001, unpaired two‐tailed *t*‐test or one‐way analysis of variance (ANOVA) with Tukey multiple comparison test.

Efficient generation and maintenance of T_RM_ cells are essential for long‐lasting immune protection in barrier tissues. In this study, we aimed to develop a novel strategy to enhance protective immunity while minimizing pathology, based on the above findings. Considering that α‐PD‐L1 treatment induces severe pulmonary pathology in hosts, we investigated whether appropriately enhancing costimulatory signals could promote the protective functions of NP_366–374_ T_RM_ cells with reduced pathology. The CD27 agonist has demonstrated promising efficacy in cancer immunotherapy; therefore, we explored its role in the maintenance of NP_366–374_ T_RM_ cells following the resolution of primary infection. PR8‐infected mice were administered the CD27 agonist starting at 21 d.p.i. While no effect of the CD27 agonist on the proliferation of NP_366–374_ T_RM_ cells was observed after two treatments at 29 d.p.i. (Figure S11A,B, Supporting Information), administration during the memory stage resulted in sustained high levels of Ki‐67 expression in these cells at 42 d.p.i. (**Figure** **6**B,C). Consequently, the CD27 agonist increased both the frequency and total cell numbers of NP_366–374_ T_RM_ cells at 60 d.p.i., with no impact on PA_224–233_ T_RM_ cells (Figure 6D,E; Figure S11C, Supporting Information). Additionally, the CD27 agonist led to elevated per‐cell expression levels of CD69 in NP_366–374_ T_RM_ cells without affecting CD103 and PD‐1 expressions (Figure 6F; Figure S11D, Supporting Information). Furthermore, the CD27 agonist enhanced T_RM_ cell‐mediated heterologous protection against influenza reinfection (Figure 6G). Given that excessive CD8^+^ T_RM_ cells in the lung may increase the risk of inflammation and fibrosis, we assessed whether the CD27 agonist contributed to pulmonary pathology. Histological analysis using Hematoxylin and Eosin staining and Masson staining of lung tissue from mice at 60 d.p.i. showed no significant differences in inflammation or tissue damage between control and agonist‐treated groups (Figure 6H; Figure S11E, Supporting Information). These data indicate that persistent activation of CD27 signaling in NP_366–374_ T_RM_ cells after the resolution of the primary influenza response promotes their long‐term maintenance without incurring pathological consequences.

Next, we evaluated whether the CD27 agonist prevents the loss of T_RM_ cells over time in a Nur77‐dependent manner. To address this, we first investigated Nur77 expression using reporter mice and observed a higher level of EGFP expression in the CD27 agonist group at 42 d.p.i. (Figure 6I,J). To clarify the relationship between NR4A1 function and the effects of the CD27 agonist on NP_366–374_ T_RM_ cells, we utilized WT and NR4A1^−/−^ mice infected with influenza PR8 and treated with the CD27 agonist from 21 to 56 d.p.i. The ability of the CD27 agonist to maintain NP_366–374_ T_RM_ cells at higher levels was abrogated in NR4A1^−/−^ mice, while the frequency and cell numbers of PA_224–233_ T_RM_ cells showed no significant differences across all groups (Figure 6L,M; Figure S11F, Supporting Information). Consistent with these observations, administration of the CD27 agonist led to an increased number of Ki‐67^+^ NP_366–374_ T_RM_ cells in WT mice compared to controls, but failed to enhance the proliferation of Ki‐67^+^ NP_366–374_ T_RM_ cells in NR4A1^−/−^ mice at 42 d.p.i. (Figure 6N,O). Furthermore, NR4A1 deficiency impaired the efficacy of the CD27 agonist in promoting heterologous immunity (Figure 6P). Additionally, we sought to determine whether the CD27 agonist could augment the effects of α‐PD‐L1 in our model. WT mice were infected with influenza PR8 and treated with the CD27 agonist and/or α‐PD‐L1 from 21 to 34 d.p.i., after which T_RM_ cell proliferation was evaluated at 35 d.p.i. We found that the combination treatment did not result in higher frequencies or total cell numbers of Ki‐67^+^ NP_366–374_ T_RM_ cells compared to either single antibody treatment group (Figure S11G,H, Supporting Information). Collectively, these data suggest that persistent activation of CD27 signaling enhances the maintenance of NP_366–374_ T_RM_ cells and host heterologous immunity without causing additional pathology.

### 2.7 Coexpression of CD27 and ICOS with PD‐1 in CD8^+^ T Cells in the Lungs of Patients with Pulmonary Fibrosis

**Figure 7:**
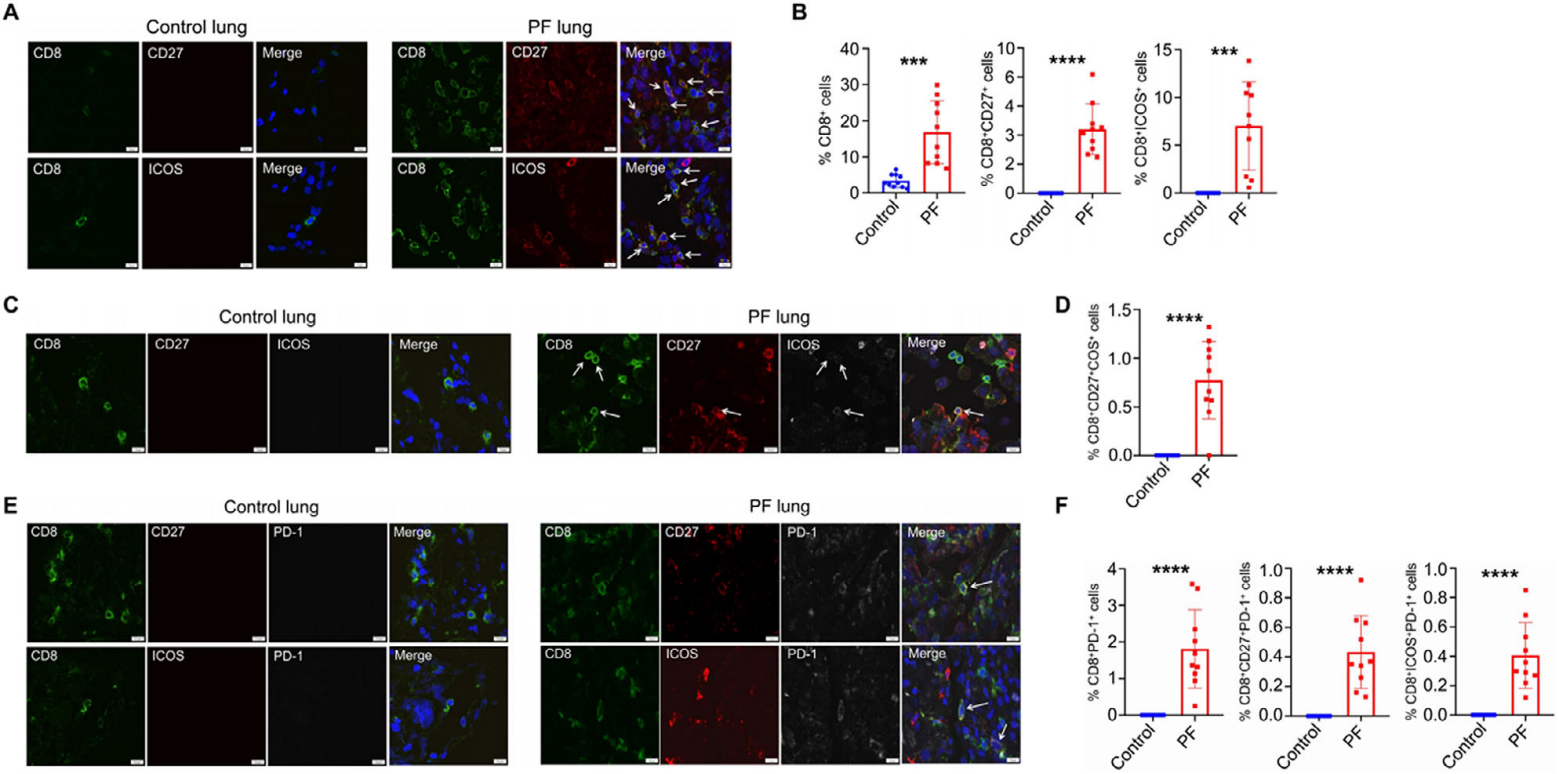
Expression of CD27 and ICOS by CD8^+^ T cells in the lungs of patients with pulmonary fibrosis. A–F) CD8, CD27, ICOS, and PD‐1 staining was performed on lung sections from control subjects (*n*=10) or patients with pulmonary fibrosis (PF) (*n*=10). A) Representative images of CD8 and CD27 or CD8 and ICOS staining. Blue indicates 4′,6‐diamidino‐2‐phenylindole (4′,6‐diamidino‐2‐phenylindol). B) Frequencies of CD8^+^, CD8^+^CD27^+^, or CD8^+^ICOS^+^ cells among DAPI^+^ cells in control and PF lungs. C) Representative images of CD8, CD27, and ICOS co‐staining. D) Frequencies of CD8^+^CD27^+^ICOS^+^ cells among DAPI^+^ cells. E) Representative images of CD8, CD27, ICOS, and PD‐1 staining. F) Frequencies of CD8^+^CD27^+^PD‐1^+^ and CD8^+^ICOS^+^PD‐1^+^ cells among DAPI^+^ cells. Mean ± SD, *** *p* < 0.001 and **** *p* < 0.0001, unpaired two‐tailed *t*‐test.

We previously reported that the frequency of CD8^+^ T cells coexpressing PD‐1 or CD103 is increased in the lungs of patients with PF, suggesting that CD8^+^ T_RM_ cells may contribute to the development of human lung fibrosis. Building on these findings, we next examined costimulatory signals among CD8^+^ T cells in the lungs affected by fibrosis. We stained lung tissues from control subjects and patients with PF for CD8 and various costimulatory molecules. Compared to control lungs, the frequencies of CD27^+^CD8^+^, ICOS^+^CD8^+^, and CD27^+^ICOS^+^CD8^+^ T cells were significantly elevated in the PF lung tissues (**Figure** **7**A–D), indicating that CD27‐ and ICOS‐mediated costimulation may be involved in the pathogenesis of human lung fibrosis. To assess the coexpression of PD‐1 with costimulatory molecules on CD8^+^ T cells, we stained human lung tissues to evaluate the presence of these receptors. A certain number of CD8^+^ T cells in human lung fibrosis displayed dual positivity for CD27^+^PD‐1^+^ or ICOS^+^PD‐1^+^ (Figure 7E,F). Overall, our data suggest that the interplay between costimulation and coinhibition in CD8^+^ T_RM_ cells may contribute to the fibrotic sequelae following acute viral infections in the human lung.

## 3 Discussion

Memory CD8^+^ T cells are long‐lived cells with stem cell‐like characteristics, equipped with cytokine responses to viral reinfections.^[^ ^4^ ^]^ Tissue‐resident memory CD8^+^ T cells play a crucial role in balancing heterosubtypic immunity and the development of fibrotic sequelae following acute influenza infection;^[^ ^5^ ^]^ however, the signals that regulate their long‐term maintenance remain poorly understood. In this study, we describe that NP_366–374_ T_RM_ cells exhibit persistent proliferative characteristics. Costimulatory molecules CD27 and ICOS are highly expressed by these T_RM_ cells and are essential for their proliferative capacity and long‐term maintenance. Importantly, CD27 and ICOS are targets of PD‐1/PD‐L1 blockade, which enhances the numbers of NP_366–374_ T_RM_ cells and promotes lung inflammation and fibrosis during the memory stage following acute viral infection. Mechanistically, the transcription factor NR4A1 plays an intrinsic role in mediating CD27 and ICOS signaling to sustain these T_RM_ cells in response to PD‐1/PD‐L1 blockade. Furthermore, CD27 and ICOS are coexpressed with PD‐1 in lung CD8^+^ T cells from patients with fibrosis, suggesting that CD27 and ICOS may serve as potential therapeutic targets for human fibrotic diseases. Our findings reveal an unexpected role for CD27 and ICOS in lung CD8^+^ T_RM_ cells.

Recently, a study reported that PD‐1‐expressing memory SARS‐CoV‐2‐specific CD8^+^ T cells elicited by infection or vaccination are not exhausted but rather functional.^[^ ^42^ ^]^ This finding is consistent with our previous observations regarding influenza infection, where we found that NP_366–374_ T_RM_ cells possess a functional status despite exhibiting an exhausted phenotype.^[^ ^5^ ^]^ In the current study, we further discovered that these T_RM_ cells exhibit a unique proliferative characteristic, persisting for weeks after the clearance of the influenza virus. A previous study demonstrated that lung CD8^+^ T_RM_ cells induced by IAV (Influenza A virus) are not maintained long term, leading to a gradual loss of protection against heterosubtypic immunity.^[^ ^33^ ^]^ Although this study indicated that the decline in lung T_RM_ cells results from an imbalance between resident memory cell apoptosis and the recruitment of circulating memory cells, this conclusion was derived using P14 TCR‐transgenic cells in the IAV model. In our prior research, we compared the phenotypes of various epitope‐specific CD8^+^ T_RM_ cells, including those specific to NP_366–374_, PA_224–233_, and PB1_703–711_ in PR8 infection, as well as ovalbumin (OVA)‐specific OT‐I TRM cells in PR8 expressing the OVA_323–339_ epitope (PR8‐OVA) infection. Our data indicated that the phenotype of transferred OT‐I T_RM_ cells resembles that of PA_224–233_ and PB1_703–711_ T_RM_ cells, particularly in displaying similarly low levels of PD‐1. Furthermore, our previous findings showed that NP_366–374_ T_RM_ cells appear to decline in number more rapidly than PD‐1^low^ PA_224–233_ T_RM_ cells. Thus, it remains to be explored whether there are additional factors preventing the long‐term maintenance of NP_366–374_ T_RM_ cells in lung tissue. Based on our findings, the persistent proliferation of NP_366–374_ T_RM_ cells at the memory stage, coupled with a gradual loss of this capacity over time, could explain their short‐lived nature in the lung. This observation raises another question: why do NP_366–374_ T_RM_ cells continue to proliferate during the memory stage? Previous studies have shown that residual NP antigen remains detectable in the lung even after viral clearance, and such prolonged antigen presentation can influence the control of local T‐cell memory.^[^ ^43^, ^44^, ^45^, ^46^, ^47^ ^]^ Our prior work also confirmed that persistent TCR/CD28 signaling is required for the long‐term maintenance of NP_366–374_ T_RM_ cells.^[^ ^5^ ^]^ Therefore, the evidence supports the notion that the persistent proliferation of NP_366–374_ T_RM_ cells identified in our study is likely dependent on continuous antigen presentation.

In our study, we found that NP_366–374_ T_RM_ cells exhibit a higher proliferative capacity compared to PA_224–233_ T_RM_ cells, yet display equivalent apoptotic susceptibility. However, the observation that NP_366–374_ T_RM_ cells decline more rapidly than their PA_224–233_ T_RM_ counterparts remains mechanistically unexplained. Previous studies have demonstrated that CXCR6, a key tissue‐residency marker, regulates the migration and compartmental distribution of CD8^+^ T_RM_ cells between the lung parenchyma and airways.^[^ ^48^ ^]^ This suggests a potential alteration in the localization of NP_366–374_ T_RM_ cells within mucosal tissues, a hypothesis that warrants further investigation. Moreover, it is increasingly recognized that T_RM_ cell residency in peripheral tissues is not static but dynamically regulated. Another plausible explanation for their accelerated disappearance is that NP_366–374_ T_RM_ cells may undergo gradual loss of costimulatory signaling in situ, leading to a concurrent decline in self‐renewal capacity and tissue‐retention potential. This functional erosion could promote their egress from the lung parenchyma into the airway lumen or even the peripheral circulation.

In this study, we aimed to identify the costimulatory and coinhibitory molecules involved in the long‐term maintenance and homeostasis of NP_366–374_ T_RM_ cells, and to elucidate their functional interactions. With regard to coinhibitory regulation, we found that only the PD‐1/PD‐L1 pathway exerts negative control over NP_366–374_ T_RM_ cells, whereas other coinhibitory receptors—such as TIM‐3, Lag‐3, and TIGIT—do not significantly impact the homeostasis of this population. Consistent with our previous findings, TIM‐3, Lag‐3, and TIGIT are expressed at low levels on NP_366–374_ T_RM_ cells compared to PD‐1, which may explain their limited regulatory capacity. The selective dependence on PD‐1‐mediated inhibition could be attributed to either the potency of PD‐1 signaling or a protective mechanism preventing excessive suppression, thereby preserving the cells’ ability to respond to secondary viral infection. Collectively, while TIM‐3, Lag‐3, and TIGIT are known to regulate exhausted CD8^+^ T cells in other disease models, their influence on T_RM_ cell homeostasis appears minimal in our influenza infection model.

The loss of CD27‐CD70 or ICOS‐ICOSL interaction impairs the proliferation of NP_366–374_ T_RM_ cells. Consequently, persistent CD27‐CD70 or ICOS‐ICOSL signaling is essential for the long‐term maintenance of these cells. Notably, we also identified other costimulatory molecules, including OX40 and CD40L, expressed by NP_366–374_ T_RM_ cells. However, blockade of OX40–OX40L or CD40–CD40L during the memory stage did not affect the persistent proliferation of NP_366–374_ T_RM_ cells. This finding does not imply that OX40–OX40L or CD40–CD40L is unimportant in regulating T_RM_ cells or in maintaining lung homeostasis. One study reported that CD40L signaling is likely involved in assisting CD4^+^ T_RM_ cells in supporting tissue B cells.^[^ ^49^ ^]^ The focus of this study is to investigate which costimulatory signals are critical for maintaining the proliferative capacity of NP_366–374_ T_RM_ cells from day 21 to day 29 following influenza infection.

Although we used α‐CD70 or α‐ICOS during the memory stage and observed a significant decrease in both the proliferative capacity and long‐term cell numbers of NP_366–374_ T_RM_ cells, we do not provide direct evidence for their intrinsic role. We blocked CD27–CD70 or ICOS–ICOSL interaction before infection; while α‐CD70 impaired the effector CD8^+^ T‐cell response, anti‐ICOS had no effect on these cells. This limitation prevented us from using CD27 KO mice to study its function in memory cells, as the absence of CD27 signaling during the effector stage leads to insufficient generation of healthy memory cells. Additionally, ICOS is essential for the optimal establishment of CD8^+^ T_RM_ cells.^[^ ^18^ ^]^ It is also important to note that CD4^+^ T cells, including Treg cells, are major cell types expressing CD27 and/or ICOS. To avoid the potential influence of CD4^+^ T‐cell‐derived CD27 and/or ICOS on the regulation of the persistent proliferation of NP_366–374_ T_RM_ cells, we depleted CD4^+^ T cells during the memory stage and found that this depletion did not alter Ki‐67 expression. More importantly, the levels of CD27 and ICOS in NP_366–374_ T_RM_ cells closely correlate with Nur77 level, cell proliferation status and IFN‐γ production, as indicated by our flow cytometry data. Therefore, our indirect evidence supports the idea that the intrinsic roles of CD27 and ICOS signaling are crucial for the long‐term maintenance of NP_366–374_ T_RM_ cells. Nonetheless, the most definitive method to clarify whether CD27 and ICOS signals play an intrinsic role in CD8^+^ T_RM_ cells in the future would be to specifically delete either gene at the memory stage using the Cre‐ERT2 system.

As the CD27–CD70 costimulation is crucial for the activation, survival, and differentiation of lymphocytes, dual PD‐1 blockade and CD27 agonism have been employed to enhance CD8^+^ T‐cell‐driven antitumor immunity.^[^ ^50^, ^51^ ^]^ ICOS is commonly expressed by various subsets of CD8^+^ and CD4^+^ T cells under different contexts. Recent study has identified a population of CXCR5^+^ CD8^+^ T cells during chronic viral infection that expresses high levels of ICOS; these cells are responsive to PD‐1 blockade and can reverse CD8^+^ T‐cell exhaustion.^[^ ^52^ ^]^ Another study demonstrated that engagement of ICOS promotes the optimal establishment of CD8^+^ T_RM_ cells in lung tissue.^[^ ^18^ ^]^ However, whether CD27 and/or ICOS play a key role in the tissue memory stage has not yet been addressed. Our work reveals, for the first time, the unexpected roles of CD27 and ICOS in the maintenance of a subset of NP_366–374_ T_RM_ cells following acute viral infection.

With regard to the detailed molecular mechanisms by which CD27/ICOS signaling maintains NP_366–374_ T_RM_ cells during the immune memory phase, this study has defined a regulatory axis: “CD27/ICOS–Nur77–Proliferation.” Nevertheless, it is likely that additional mechanisms contribute to this process. Notably, our RNA‐seq analysis revealed significant differences in the enrichment of the FOXO (Forkhead box O) signaling pathway between the control and α‐ICOS treatment groups. Previous studies have demonstrated that ICOS signaling promotes CD4^+^ T follicular helper (Tfh) cell differentiation by inactivating FOXO1 and subsequently suppressing KLF2 expression.^[^ ^53^ ^]^ Moreover, ICOS‐mediated downregulation of KLF2 has been shown to facilitate the establishment of CD8^+^ T_RM_ cells. These findings collectively highlight the critical role of the ICOS–FOXO signaling in T‐cell differentiation and long‐term maintenance across diverse immunological contexts. Therefore, further investigation into this pathway will be an important direction for future research in our model system.

The mechanism by which PD‐1/PD‐L1 blockade reinvigorates T‐cell exhaustion has become a prominent topic in the field. PD‐1 in CD8^+^ T cells has been shown to recruit the phosphatase SHP2, which suppresses proximal TCR/CD28 signaling‐mediated cell activation.^[^ ^30^, ^54^ ^]^ Our previous work also demonstrated that the reinvigoration of NP_366–374_ T_RM_ cells mediated by α‐PD‐L1 is dependent on CD28/B7 signaling.^[^ ^5^ ^]^ However, whether additional targets of α‐PD‐1 exist in CD8^+^ T cells remains unclear. Considering that most α‐PD‐1‐responsive cells usually express high levels of costimulatory molecules, it is reasonable to assume that other costimulatory molecules could serve as targets of PD‐1/PD‐L1 blockade to reinvigorate CD8^+^ T cells. Our data revealed potential novel targets of α‐PD‐L1 for rejuvenating CD8^+^ T cells. Nonetheless, further mechanistic insights into how PD‐1 inhibits CD27 and/or ICOS activity are needed in the future.

NR4A1 has been previously reported as a key mediator of T‐cell dysfunction. A recent study further identified that NR4A1 plays a crucial role in regulating progenitor‐like exhausted (Tpex) T cells in tumors, which are important for long‐term tumor or pathogen control and serve as the main responders in immunotherapy.^[^ ^55^ ^]^ Notably, NR4A1 deletion has been shown to improve tumor control.^[^ ^55^ ^]^ In our study, the findings regarding NR4A1's role in exhausted T cells within tumors present both similarities and differences. The similarity lies in the fact that NR4A1 is essential for maintaining high levels of PD‐1 on NP_366–374_ T_RM_ cells during acute infections, while the difference is that NR4A1 is required for the long‐term maintenance of these cells. These data demonstrate that NR4A1 has multiple functions in CD8^+^ T cells under different contexts.

Idiopathic pulmonary fibrosis (IPF) is a progressive disease with a median survival of 3–5 years.^[^ ^56^ ^]^ T cells, particularly Th cells, have been found to promote lung fibrosis in animal models.^[^ ^57^, ^58^, ^59^ ^]^ ICOS signaling is essential for Interleukin‐5 (IL‐5)  production by ILC2s, which protects against acute exacerbations of lung injury and fibrosis.^[^ ^60^ ^]^ In human, the population of CD103^+^ CD4^+^ T cells were significantly increased in the airway of patients with fibrotic lung disease.^[^ ^61^ ^]^ The declines in ICOS and CD28 expressions in CD4^+^ T cells are associated with decreased lung function in IPF.^[^ ^62^ ^]^ Recent studies have shown that memory CD8^+^ T cells likely play a critical role in acute lung injury and fibrosis following respiratory viral infections;^[^ ^5^, ^6^, ^63^ ^]^ however, it remains unclear whether costimulation in memory CD8^+^ T cells is associated with the progression of lung injury and fibrosis. Our observations of pulmonary fibrosis lung tissues revealed an enrichment of CD27^+^PD‐1^+^ and ICOS^+^PD‐1^+^ CD8^+^ T cells, suggesting that CD27 and ICOS costimulation may be involved in lung fibrosis. It should also be noted that only a small fraction of PD‐1^+^ cells exist among the CD27^+^ CD8^+^ or ICOS^+^ CD8^+^ T cells, suggesting that the loss of PD‐1 may potentially lead to the overactivation of these CD8^+^ T cells through CD27 or ICOS signaling. Furthermore, IPF is strongly associated with advanced age, typically affecting individuals aged 70–75, and is rare in those under 50.^[^ ^64^, ^65^ ^]^ Notably, T cells gradually lose CD28 expression in the elderly.^[^ ^66^, ^67^ ^]^ In addition to CD28, the expression of other costimulatory molecules, such as CD27, on T cells declines with aging. Therefore, before targeting CD27 and ICOS as potential therapeutic strategies for pulmonary fibrosis driven by aberrant T‐cell expansion, it is crucial to evaluate the expression levels of these molecules on infiltrating T cells in the context of age‐related immune changes.

Some limitations of this study are worth noting. In our research, we have only examined the function of CD27 and ICOS as targets of PD‐1/PD‐L1 signaling in the lung following acute infection. Notably, NP_366–374_ T_RM_ cells identified in our acute IAV model are exhausted‐like T_RM_ cells, which differ from the truly exhausted T cells observed in tumor or chronic viral models. Whether CD27 and ICOS, along with other costimulatory molecules, play similar roles in other mucosal tissues infected by distinct pathogens warrants further investigation. Additionally, the acquisition of exhaustion by CD8^+^ T cells is a complex process involving numerous transcription factors, including NR4A1, Tox, Bcl6, and others.^[^ ^40^, ^41^, ^68^, ^69^, ^70^, ^71^ ^]^ While we highlighted NR4A1's role in mediating the long‐term maintenance and abnormal expansion of NP_366–374_ T_RM_ cells, the relationships and mechanisms of interaction among these key transcription factors remain unknown. Further studies are necessary to unravel how the regulatory network guides the formation of NP_366–374_ T_RM_ cells and how these cells intricately collaborate with costimulation and coinhibition to maintain tissue homeostasis.

## 4 Experimental Section

### Mouse and Infection

Healthy adult C57BL/6 mice, weighing 18–25 g and aged 8–10 weeks, were housed in cages with access to free water and food. These mice were purchased from the Fourth Military Medical University. CD8α^−/−^ and NR4A1^−/−^ mice were originally purchased from Cyagen Biosciences (Suzhou), Nur77–EGFP mice were originally purchased from the Jackson Laboratory and bred in house. Sex‐matched and age‐matched 9–12 week old mice of both sexes were used in the experiments. All animal experiments were approved by Animal Care and Use Committees of the Fourth Military Medical University (No. IACUC‐20230524). For influenza virus infection, influenza PR8 virus (≈600 pfu per mouse unless stated in the text in the primary infection) or X31 virus (≈1×10^4^ or ≈2×10^4^ pfu per mouse in the secondary infection as indicated in the text) was diluted in fetal bovine serum‐free Dulbecco's modified Eagle's medium (Corning) on ice and inoculated into anesthetized mice via the intranasal route. To facilitate viral entry into the lungs, gentle pressure was applied to the jaw while administering the virus into the nasal cavity.

### Human Lung Tissue Specimen

Surgical lung biopsy specimens from patients with biopsy diagnosis (clinically PF) of common interstitial pneumonia (UIP) were obtained from the Department of Pathology of Xijing Hospital. Control lung tissue was obtained from surgical biopsy specimens of patients with benign indications (all benign pulmonary nodules), with sections taken from normal lung tissue surrounding the resected nodules. It was important to note that there was no evidence of interstitial lung disease in the selected control group. The use of human lung tissue specimens in this study was approved by the Institutional Review Committee of the Fourth Military Medical University (Grant No. KY20243064‐1).

### Immunofluorescence Staining

CD8/CD27/ICOS/PD‐1 staining was performed on formalin‐fixed, paraffin‐embedded (FFPE) lung tissue sections. The paraffin sections underwent sequential treatment with Environmental Dewaxing Solutions I, II, and III (g1128, Servicebio) for 10 min each, followed by three washes in absolute ethanol I, II, and III for 5 min each, and finally rinsed in distilled water. Following antigen retrieval, an immunohistochemical pen (G6100, Servicebio) was used to delineate a barrier around the tissue. After blocking with 3% bovine serum albumin (BSA; GC305006, Servicebio), the primary antibody, rabbit anti‐CD8 (Servicebio), prepared in sterile PBS (G4250, Servicebio), was applied and incubated overnight at 4 °C. The corresponding Horseradish Peroxidase (HRP)‐labeled goat anti‐mouse IgG (Servicebio) was then added and incubated for 50 min at room temperature. Tyramide Signal Amplification (TSA) (488) was applied next and incubated at room temperature in the dark for 10 min. The tissue sections were placed in a repair box containing pH 6.0 citric acid repair solution (G1202, Servicebio) and heated in a microwave oven at boiling temperature for 10 min before serum blocking. Subsequently, rabbit anti‐CD27 (GB11583, Servicebio) or rabbit anti‐ICOS (ab264644, Abcam) was added and incubated overnight at 4 °C. The corresponding HRP‐labeled goat antirabbit IgG (Servicebio) was then added and incubated for 50 min at room temperature. TSA (555) was subsequently applied and incubated at room temperature in the dark for another 10 min. After microwave treatment, PD‐1 (#86 163, CST) was introduced and incubated overnight at 4 °C. The corresponding HRP‐labeled goat antimouse IgG (Servicebio) was added and incubated for 50 min at room temperature, followed by TSA (647) incubation at room temperature in the dark for 10 min. Following incubation, the slides were washed three times with shaking using Tris‐buffered saline with Tween 20 (G0004, Servicebio) for 5 min each. The cell nuclei were counterstained with 4′,6‐diamidino‐2‐phenylindole (DAPI, G1012, Servicebio) for 10 min in the dark at room temperature. An Autofluorescence Quencher (G1221, Servicebio) was added for 5 min and rinsed with running water for 10 min. After washing and slightly drying, the sections were sealed with an antifluorescence quenching sealing agent (G1401, Servicebio). Panoramic scanning of the sections was conducted using an upright fluorescence microscope (NIKON ECLIPSE C1) and a scanner (Pannoramic MIDI).

### RNA‐seq and Data Analysis

The sorted cells were centrifuged at 1600 rpm for 5 min, and the supernatant was discarded. Approximately 200 µL of Trizol lysate was added until the solution became clear and nonviscous, after which the sample was sent to LC Bio Technologies (Hangzhou) for Smart‐Seq processing. The SMART‐Seq v4 Ultra Low Input RNA Kit (Clontech, Japan) was used to prepare the low‐input library following the outlined procedures: preparing the complementary DNA (cDNA) library, performing cDNA purification and size selection (150–300 bp), and preparing the sequencing‐ready library by adding i5/i7 adaptors using Polymerase Chain Reaction. Library quality control was conducted to assess the size and concentration of the library using the Agilent 2100 Bioanalyzer. Sequencing was performed on Illumina platforms using a 2×150 bp paired‐end sequencing protocol. EdgeR (https://bioconductor.org/packages/release/bioc/html/edgeR.html) was employed to visualize the differential expression results using a gene heat map, scatter plot, volcano plot, and principal component analysis. Expression levels of all transcripts were estimated, and FPKM (FPKM = [total exon fragments/mapped reads (millions) × exon length (kB)]) was calculated. Differentially expressed messenger RNA were selected based on a fold change of greater than 2 or less than 0.5, with a *p*‐value less than 0.05 as determined by EdgeR. Functional analysis of differentially expressed genes included Gene Ontology (GO) enrichment analysis and Kyoto Encyclopedia of Genes and Genomes (KEGG) pathway enrichment analysis.

### Histopathological Analysis of the Lungs

Following euthanasia, 10 mL of PBS was perfused into the mice through the right ventricle. The lungs were carefully instilled with 10% paraformaldehyde (PF) and left inflated for 1 min before excising the lobe and placing it in 10% PF for 48 h, after which it was moved to 70% ethanol. The samples were then sent to the Servicebio Pathology Laboratory (Servicebio Technology, Wuhan). The corresponding tissue sections were prepared according to the experimental standard operating procedures (SOP) of Servicebio, including pathological tissue sampling and fixation, embedding, paraffin sectioning, and frozen sections. They were sliced into 3–4 µm sections for staining with hematoxylin and eosin and Masson's trichrome.

The hematoxylin and eosin staining procedures included immersing the paraffin sections sequentially in an environmentally friendly dewaxing transparent liquid I for 20 min, followed by liquid II for 20 min, anhydrous ethanol I for 5 min, anhydrous ethanol II for 5 min, and 75% ethyl alcohol for 5 min, followed by a rinse with tap water. The frozen sections were retrieved from the −20 °C refrigerator, allowed to reach room temperature, fixed with a tissue fixation solution for 15 min, and rinsed with running water. The sections were treated with High definition constant staining pretreatment solution for 1 min, immersed in hematoxylin solution for 3–5 min, and then rinsed with tap water. Subsequently, the sections were treated with hematoxylin differentiation solution and rinsed again with tap water. The sections were then treated with hematoxylin bluing solution, rinsed with tap water, placed in 95% ethanol for 1 min, and stained with eosin for 15 s. The sections were then immersed in absolute ethanol I for 2 min, absolute ethanol II for 2 min, absolute ethanol III for 2 min, normal butanol I for 2 min, normal butanol II for 2 min, xylene I for 2 min, and xylene II for 2 min, and finally sealed with neutral gum.

The Masson's trichrome staining procedures involved soaking the slices in Masson A overnight, followed by rinsing with tap water. Masson B and C were mixed in a 1:1 ratio to prepare the Masson solution. The slices were stained with this solution for 1 min and then rinsed with tap water. Differentiation was achieved using 1% hydrochloric acid alcohol for several seconds, followed by rinsing with tap water. The slices were soaked in Masson D for 6 min, rinsed with tap water, and then treated with Masson E for 1 min. The slices were slightly drained and immersed in Masson F for 2–30 s. They were rinsed with 1% glacial acetic acid before dehydration with two cups of anhydrous ethanol. For clearing and sealing, the slides were soaked in 100% ethanol for 5 min, xylene for 5 min, and finally sealed with neutral gum. Microscopic inspection (Nikon Eclipse 100), image acquisition, and analysis (Nikon D3‐U3) were performed for the tissue sections.

### Labeling of Circulating CD8^+^ T Cells

Anti‐CD45 Percp5.5 antibodies were diluted in 200 µL of sterile PBS and then injected intravenously into mice to distinguish between circulating and resident CD45^+^ cells. The mice were euthanized, and lung tissues were collected 5 min post‐antibody injection. Lung tissues were dissociated using the gentleMACS (Miltenyi Biotec) at 37 °C for 30 min. Lung circulating CD45^+^ cells were identified as those that were antibody positive, while lung‐resident T cells were defined as those that were antibody negative.

### Flow Cytometry

Fluorescence‐activated cell sorting (FACS) antibodies were primarily obtained from BD Pharmingen and BioLegend. The clone numbers of these antibodies utilized in this study were as follows: CD8α (KT15), CD45 (30‐F11), Ki‐67 (SolA15), CD69 (H1.2F3), CD103 (2E7), PD‐1 (29F.1A12), and IFN‐γ (XMG1.2). The dilution rates for these antibodies were 1:200 for surface staining and 1:100 for intracellular staining. H‐2Db‐NP_366–374_ and H‐2Db‐PA_224–233_ tetramers were procured from MBL, with a dilution of 1:50 for surface staining. After staining, cells were analyzed using the SA3800 Spectroscopy Analyzer System (Sony Biotechnology), and the data were processed with FlowJo software (version 10.9.0, Tree Star).

### Intracellular Staining

Lung cells in suspension were collected and stained with the specified surface markers at 4 °C in the dark for 30 min. Cells were then washed twice with FACS buffer (PBS, 2 mm Ethylenediaminetetraacetic acid, 1% Fetal Bovine Serum, and 0.09% sodium azide) and subsequently fixed and permeabilized using the Foxp3 transcription factor staining buffer set (eBioscience). After fixation, cells were stained with antibodies against Ki‐67 or IFN‐γ for 1 h at room temperature in the dark and washed twice with per wash (eBioscience) prior to flow cytometry acquisition.

### FTY720 Treatment

In the influenza rechallenge experiments, mice received daily intraperitoneal injections of FTY720 (1 mg kg^−1^; Cayman) starting three days before rechallenge and continuing until the mice were sacrificed for TRM analysis.

### Ab Blockade or Depletion In Vivo

Anti‐CD70, anti‐ICOS, anti‐CD40L, anti‐OX40L, anti‐PD‐L1, anti‐TIM‐3, anti‐LAG‐3, anti‐TIGIT, anti‐CD27, anti‐CD4, and control antibodies were sourced from Bio X Cell. For the effector T‐cell experiment, mice were infected with influenza and received intraperitoneal injections of control or blocking antibodies (300 µg per mouse) every 3 days starting 1 day before infection. In the T_RM_ cell experiment, influenza‐infected mice received an initial intraperitoneal injection of control or blocking antibodies (500 µg per mouse) at 21 d.p.i., followed by injections of 300 µg per mouse every 4 days thereafter. For the anti‐CD27 agonistic antibody, mice were administered 500 µg per mouse every five days starting at 21 d.p.i. For the CD4 depletion experiment, mice received intraperitoneal injections of 500 µg per mouse every 4 days starting at 21 d.p.i.

### Bone Marrow Chimera

To create an NR4A1‐specific deficiency in CD8^+^ T cells in mice, recipient CD45.1 mice were administered Busulfan (Sigma) at a dose of 100 mg kg^−1^ for five consecutive days. The mice were then reconstituted with a mixture of WT and CD8α^−/−^ bone marrow cells at a ratio of 3:1 with NR4A1^−/−^ bone marrow cells. Mice were allowed to rest for 8 weeks before being infected with influenza. The mice were euthanized at the indicated time points following influenza infection for experimentation.

### Peptide Restimulation In Vitro

Lung cell suspensions were restimulated with NP_366–374_ peptide (1 µg mL^−1^) (AnaSpec) for 5 h in the presence of Monensin Solution (eBioscience). After restimulation, cells were first stained with surface markers and then fixed and permeabilized using a Fixation/Permeabilization Kit to stain IFN‐γ.

### Statistical Analysis

A nonpaired two‐tailed Student's *t*‐test was employed to compare the means of two groups. For comparisons involving more than two groups, one‐way analysis of variance (ANOVA) followed by Tukey's multiple comparison test was utilized. Statistical analyses were conducted using Prism 6 software. *p < 0.05, **p < 0.01, ***p < 0.001, and ****p < 0.0001.

## Author Contributions

Y.Y.C., Z.Z.W., and L.Q.S. contributed equally to this work. Z.W. and L.S. conceived the project and designed experiments. Y.Y.C., Z.Z.W., Q.Q.C., L.Q.S., H.Z., T.P., J.L.P., T.S., H.E.X., and J.L. performed experiments and analyzed data. J.L.P., L.Q.S., S.Z., X.L., F.K., Y.Z.N., and F.Z. provided important reagents and funding. Z.W., L.S., Z.Z.W., and Y.Y.C. wrote the paper.

## Conflict of Interest

The authors declare no conflict of interest.

## Supporting information



Supporting Information

## Data Availability

Research data are not shared.
