# Supplementary material for: CD27 and ICOS as Targets of PD‐1/PD‐L1 Signaling to Regulate Resident Memory CD8+ T‐Cell‐Mediated Pulmonary Protection and Pathology
Source: Adv Sci (Weinh). 2025 Nov 20;13(7):e12452. doi: 10.1002/advs.202512452 (PMC12866844; doi:10.1002/advs.202512452)
Supplement: Supplementary file 1 — Supporting Information [file ADVS-13-e12452-s001.docx]

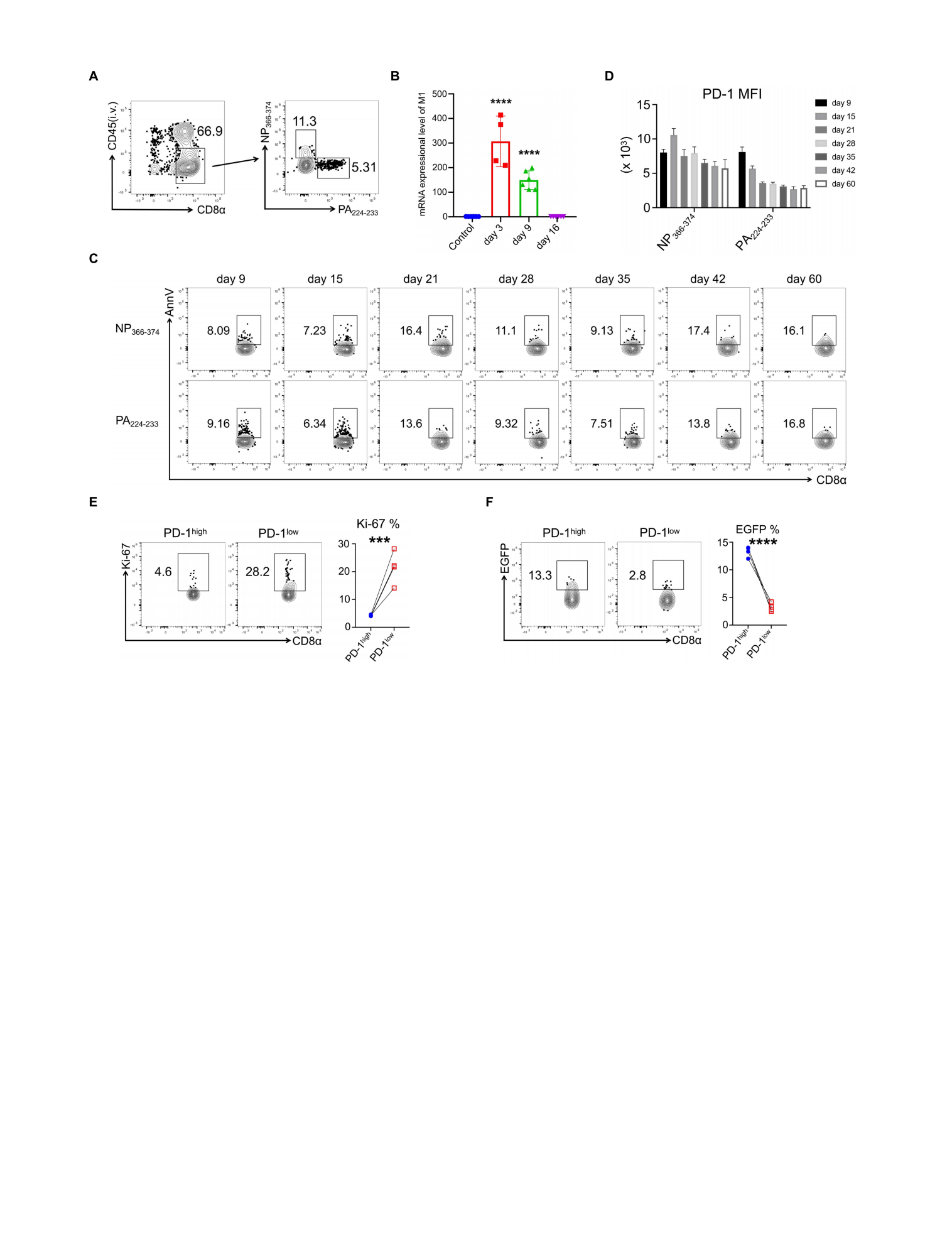


**Supplementary Figure 1**. **Proliferative characteristics of NP_366-374_ CD8^+^ T_RM_ cells.** (**A**) Gating strategy of sorted lung NP_366-374_ and PA_224-233_ T_RM_ cells. (**B**) Quantification of viurs gene expression in the lungs at the indicated d.p.i. (**C**) Representative plots of lung AnnV^+^ NP_366-374_ or PA_224-233_ CD8^+^ T cells assessed by flow cytometry at the indicated d.p.i. (**D**) MFI of PD-1 on lung NP_366-374_ and PA_224-233_ T_RM_ cells at the indicated d.p.i. (**E**) Ki-67 expression of PD-1^high^ or PD-1^low^ lung NP_366-374_ T_RM_ cells was assessed by flow cytometry at 28 d.p.i. (**F**) Nur77-EGFP expression of PD-1^high^ or PD-1^low^ lung NP_366-374_ T_RM_ cells was assessed by flow cytometry at 28 d.p.i.


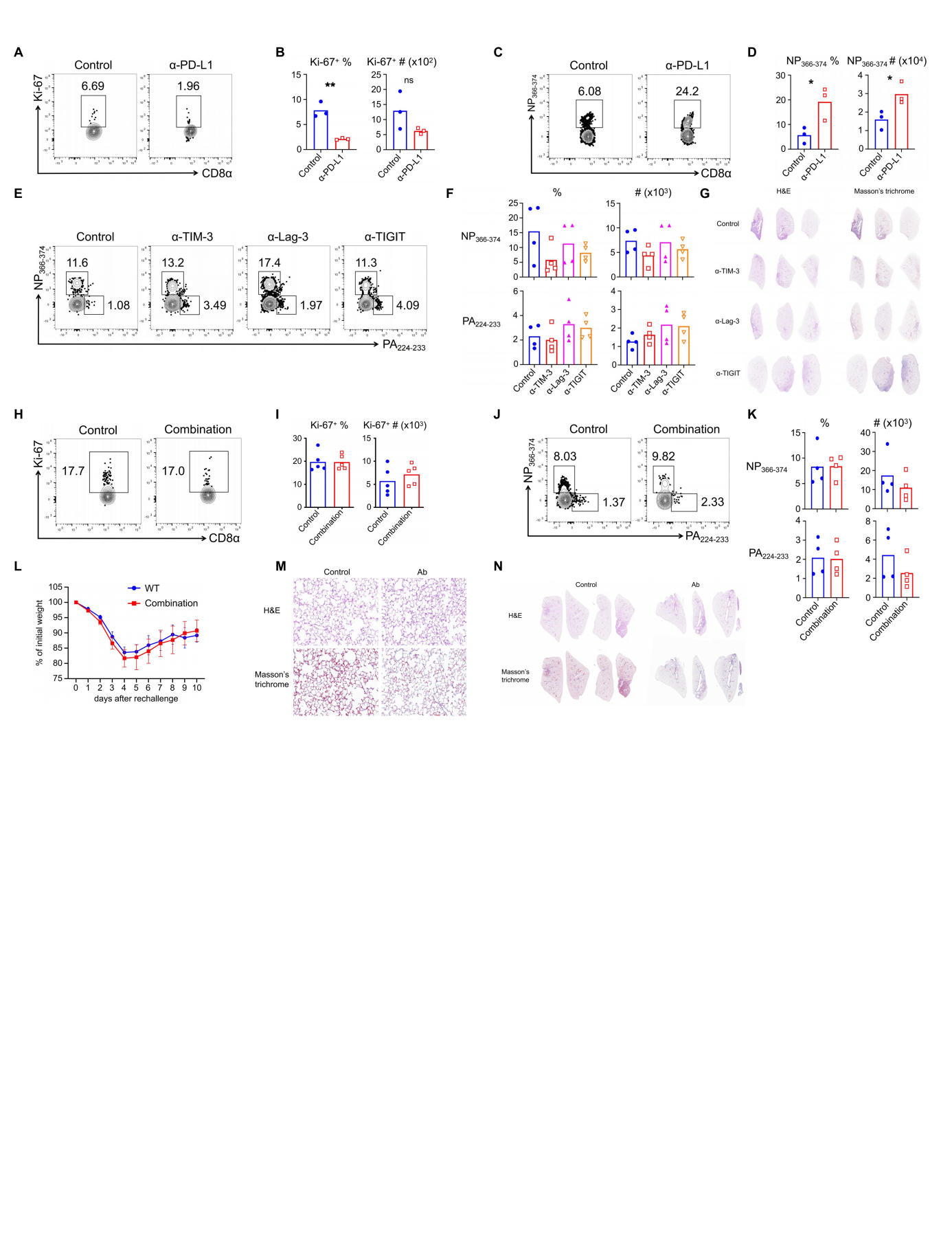


**Supplementary Figure 2**. **Coinhibitory mechanisms in the regulation of NP_366-374_ CD8^+^ T_RM_ cells.** (**A** to **D**) WT C57BL/6 mice were infected with influenza PR8 and received either control IgG or α-PD-L1 from 21 to 37 d.p.i. (A and B) Representative plots, frequencies, and total cell numbers of Ki-67^+^ NP_366-374_ T_RM_ cells at 42 d.p.i. (C and D) Representative plots, frequencies, and total cell numbers of NP_366-374_ T_RM_ cells at 42 d.p.i. (**E** to **G**) WT C57BL/6 mice were infected with influenza PR8 and received control IgG, α-TIM-3, α-Lag-3 or α-TIGIT from 21 to 37 d.p.i. (E and F) Representative plots, frequencies, and total cell numbers of NP_366-374_ and PA_224-233_ T_RM_ cell at 42 d.p.i. (G) Lung pathology were assessed at 60 d.p.i. (**H** to **N**) WT C57BL/6 mice were infected with influenza PR8 and received control IgG or a combination of α-TIM-3, α-Lag-3 and α-TIGIT from 21 to 25 or 37 d.p.i. (H and I) Representative plots, frequencies, and total cell numbers of Ki-67^+^ NP_366-374_ T_RM_ cells at 29 d.p.i. (J and K) Representative plots, frequencies, and total cell numbers of NP_366-374_ and PA_224-233_ T_RM_ cell at 42 d.p.i. (L) All mice were rechallenged with X31 (2×10^4^ pfu) at 42 d.p.i. in the presence of FTY720. Percentages of original body weight following rechallenge were assessed daily. (M and N) Lung pathology were assessed at 60 d.p.i. Representative of three experiments (n = 3 to 5). Data are mean ± SD. *P < 0.05, **P < 0.01, unpaired two-tailed t test.


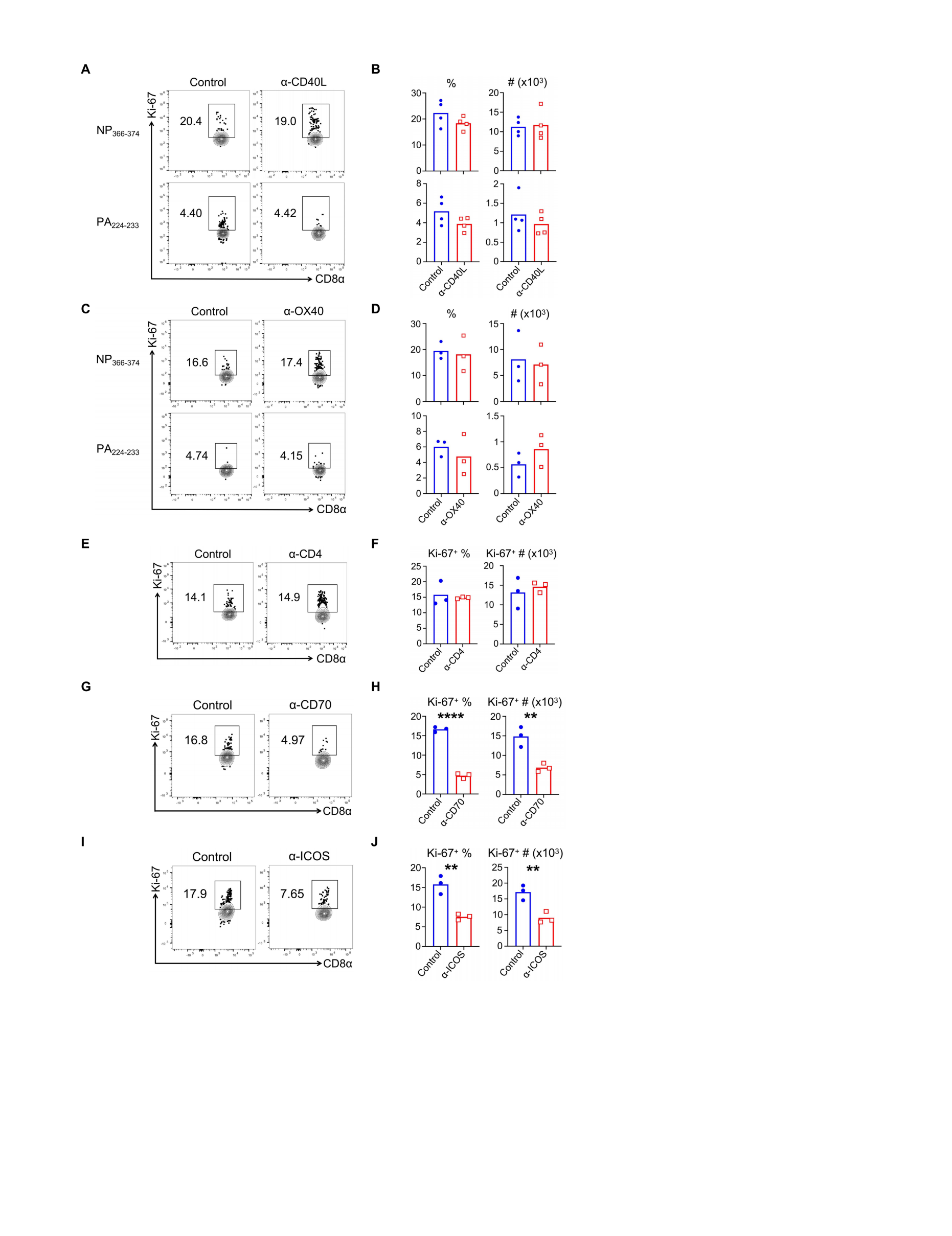


**Supplementary Figure 3. Costimulatory mechanisms in the regulation of NP_366-374_ CD8^+^ T_RM_ cells.** (**A** to **D**) WT C57BL/6 mice were infected with influenza PR8 and received either control IgG, anti-CD40L (α-CD40L), or anti-OX40 (α-OX40) from 21 to 25 d.p.i. Representative plots, frequencies, and total cell numbers of Ki-67^+^ NP_366-374_ T_RM_ cells at 29 d.p.i. (**E** and **F**) WT C57BL/6 mice were infected with influenza PR8 and received either control IgG or anti-CD4 (α-CD4) depletion antibody from 21 to 25 d.p.i. Representative plots, frequencies, and total cell numbers of Ki-67^+^ NP_366-374_ T_RM_ cells at 29 d.p.i. (**G** to **J**) WT C57BL/6 mice were infected with influenza PR8 and received α-CD4 depletion antibody from 20 to 25 d.p.i. Then mice were received either control IgG, α-CD70, or α-ICOS from 21 to 25 d.p.i. Representative plots, frequencies, and total cell numbers of Ki-67^+^ NP_366-374_ T_RM_ cells at 29 d.p.i. Representative of three experiments (n = 3 to 4). Data are mean ± SD. Unpaired two-tailed t test.


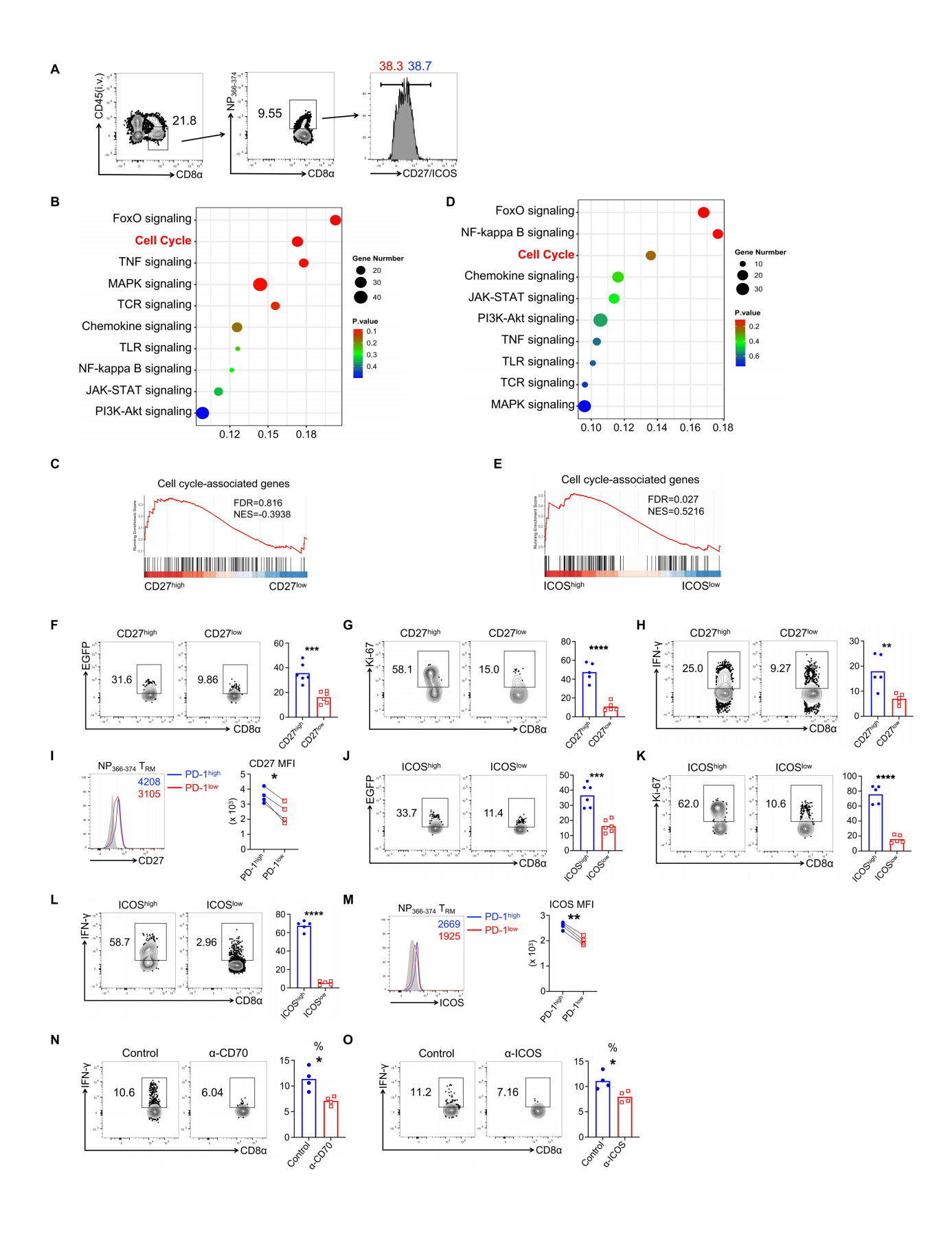


**Supplementary Figure 4. Expression of CD27 and ICOS correlates with cell proliferation, Nur77 levels and cytokine production.** (**A**) Gating strategy for discriminating between high and low levels of CD27 or ICOS expression in NP_366-374_ T_RM_ cells. (**B**) Go pathway enrichment analysis of NP_366-374_ T_RM_ cells based on high versus low expression of CD27. (**C**) GSEA showing positive enrichment of cell cycle-associated genes in CD27^high^ NP_366-374_ T_RM_ cells. (**D**) Go pathway enrichment analysis of NP_366-374_ T_RM_ cells based on high versus low expression of ICOS. (**E**) GSEA showing positive enrichment of cell cycle-associated genes in ICOS^high^ NP_366-374_ T_RM_ cells. (**F** and **J**) Nur77-EGFP mice were infected with influenza PR8. Representative plots and frequencies of EGFP^+^ NP_366-374_ T_RM_ cells based on CD27 (F) or ICOS (I) expression at 28 d.p.i. (**G** and **K**) WT C57BL/6 mice were infected with influenza PR8. Representative plots and frequencies of Ki-67^+^ NP_366-374_ T_RM_ cells based on CD27 (G) or ICOS (J) expression at 28 d.p.i. (**H** and **L**) WT C57BL/6 mice were infected with influenza PR8. Representative plots and frequencies of IFN-γ^+^ NP_366-374_ T_RM_ cells based on CD27 (H) or ICOS (K) expression at 28 d.p.i. (**I** and **M**) WT C57BL/6 mice were infected with influenza PR8. MFI of CD27 (I) or ICOS (M) on PD-1^high^ or PD-1^low^ NP_366-374_ T_RM_ cells at 28 d.p.i. (**N** and **O**) WT C57BL/6 mice were infected with influenza PR8 and received either control IgG, α-CD70, or α-ICOS from 21 to 35 d.p.i. Representative plots and frequencies of IFN-γ^+^ NP_366-374_ T_RM_ cells at 35 d.p.i. Representative of three experiments (n = 4 to 6) for (F to O). Data are mean ± SD. **P < 0.01, ***P < 0.001, ****P < 0.0001, unpaired two-tailed t test.


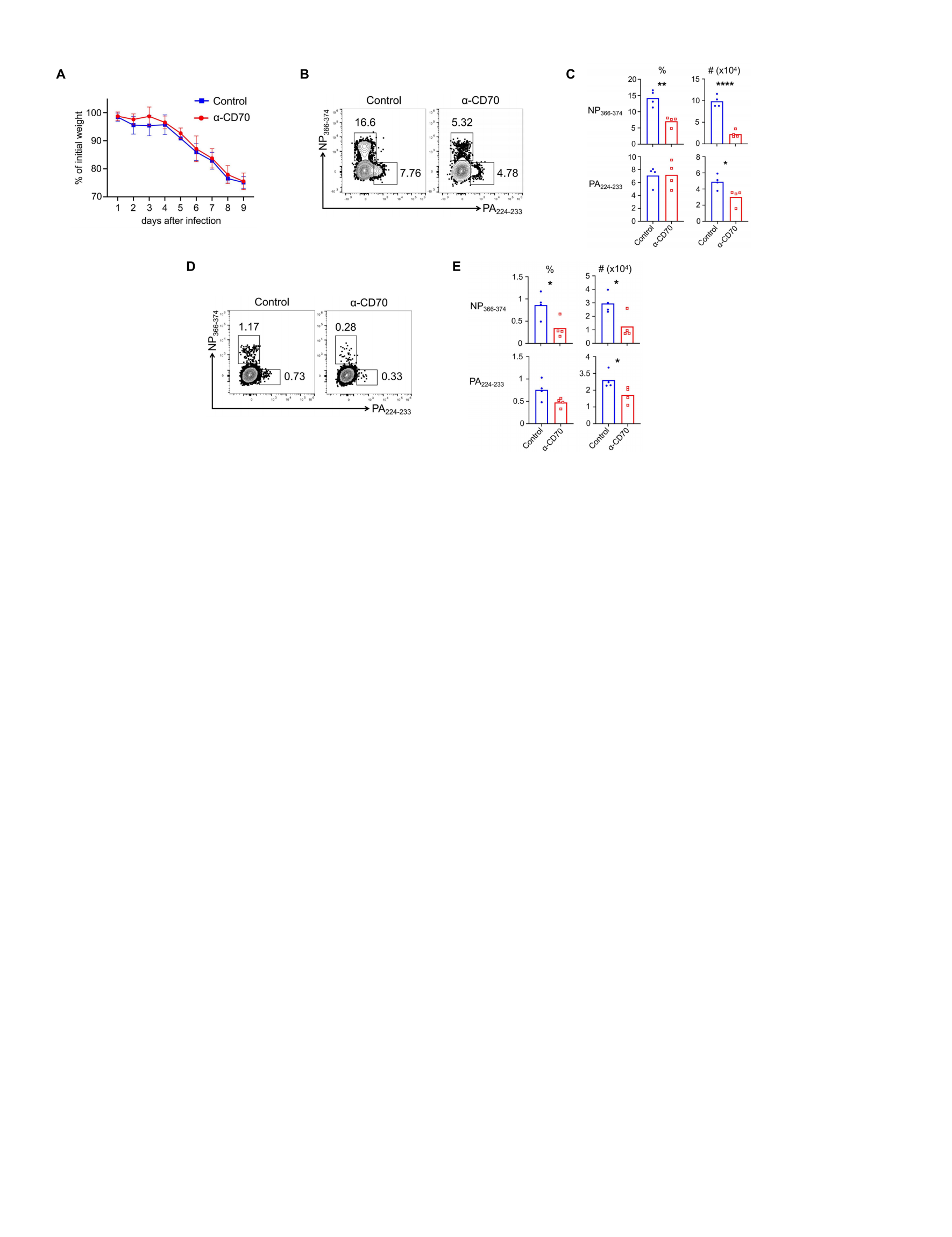


**Supplementary Figure 5. CD27-CD70 interaction in the effector CD8^+^ T cells response following influenza infection.** (**A** to **E**) WT C57BL/6 mice were infected with influenza PR8 and received either control IgG or α-CD70 from day -1 to 9 d.p.i. (A) Percentages of original body weight after primary infection were assessed daily. (B and C) Representative plots, frequencies, and total cell numbers of NP_366-374_ and PA_224-233_ CD8^+^ T cells in the lungs. (D and E) Representative plots, frequencies, and total cell numbers of NP_366-374_ and PA_224-233_ CD8^+^ T cells in the spleen. Representative of three experiments (n = 4). Data are mean ± SD. *P < 0.05, **P < 0.01, ****P < 0.0001, unpaired two-tailed t test.


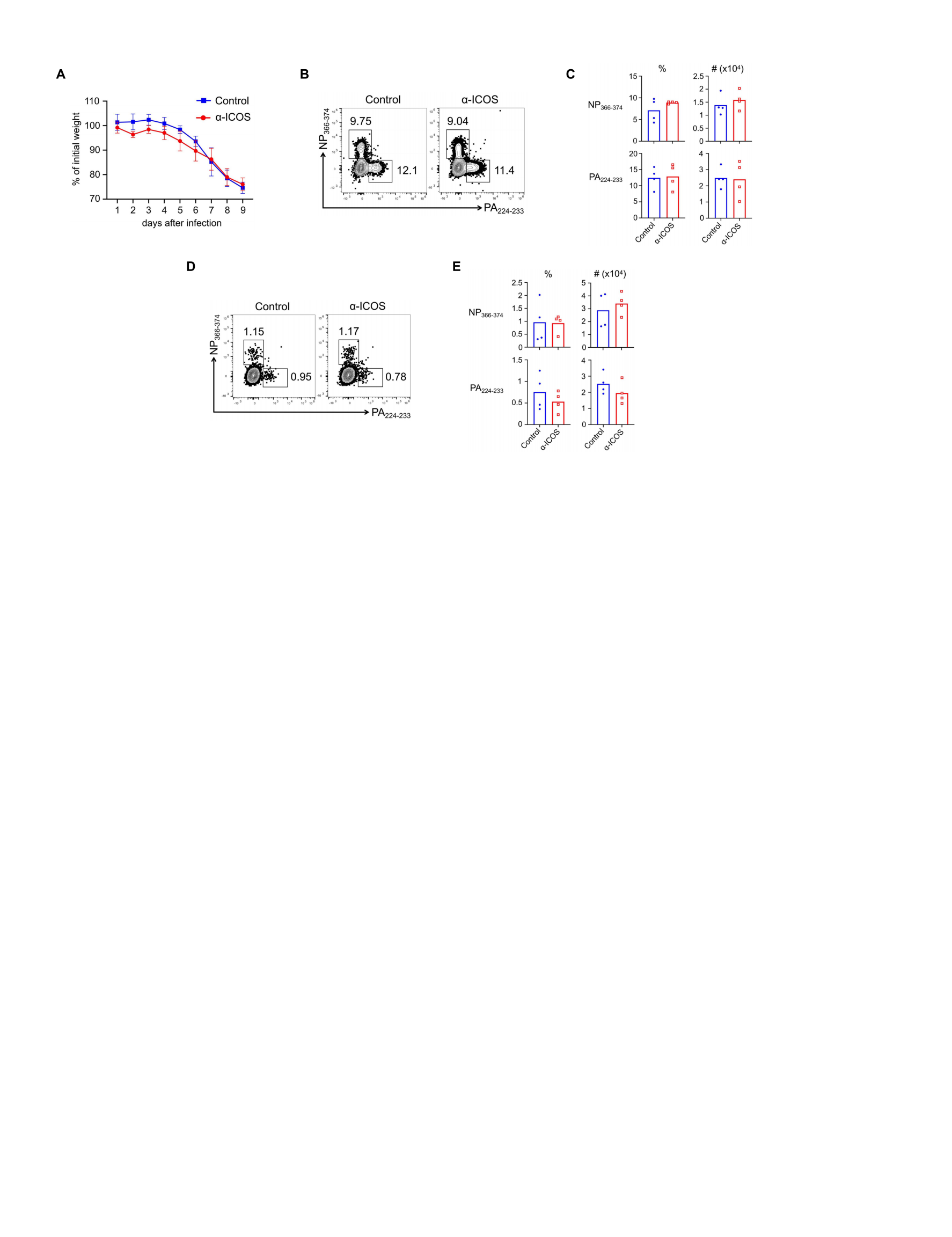


**Supplementary Figure 6**. **ICOS-ICOSL interaction in the effector CD8^+^ T cells response following influenza infection.** (**A** to **E**) WT C57BL/6 mice were infected with influenza PR8 and received either control IgG or α-ICOS from day -1 to 9 d.p.i. (A) Percentages of original body weight after primary infection were assessed daily. (B and C) Representative plots, frequencies, and total cell numbers of NP_366-374_ and PA_224-233_ CD8^+^ T cells in the lungs. (D and E) Representative plots, frequencies, and total cell numbers of NP_366-374_ and PA_224-233_ CD8^+^ T cells in the spleen. Representative of three experiments (n = 4). Data are mean ± SD. Unpaired two-tailed t test.


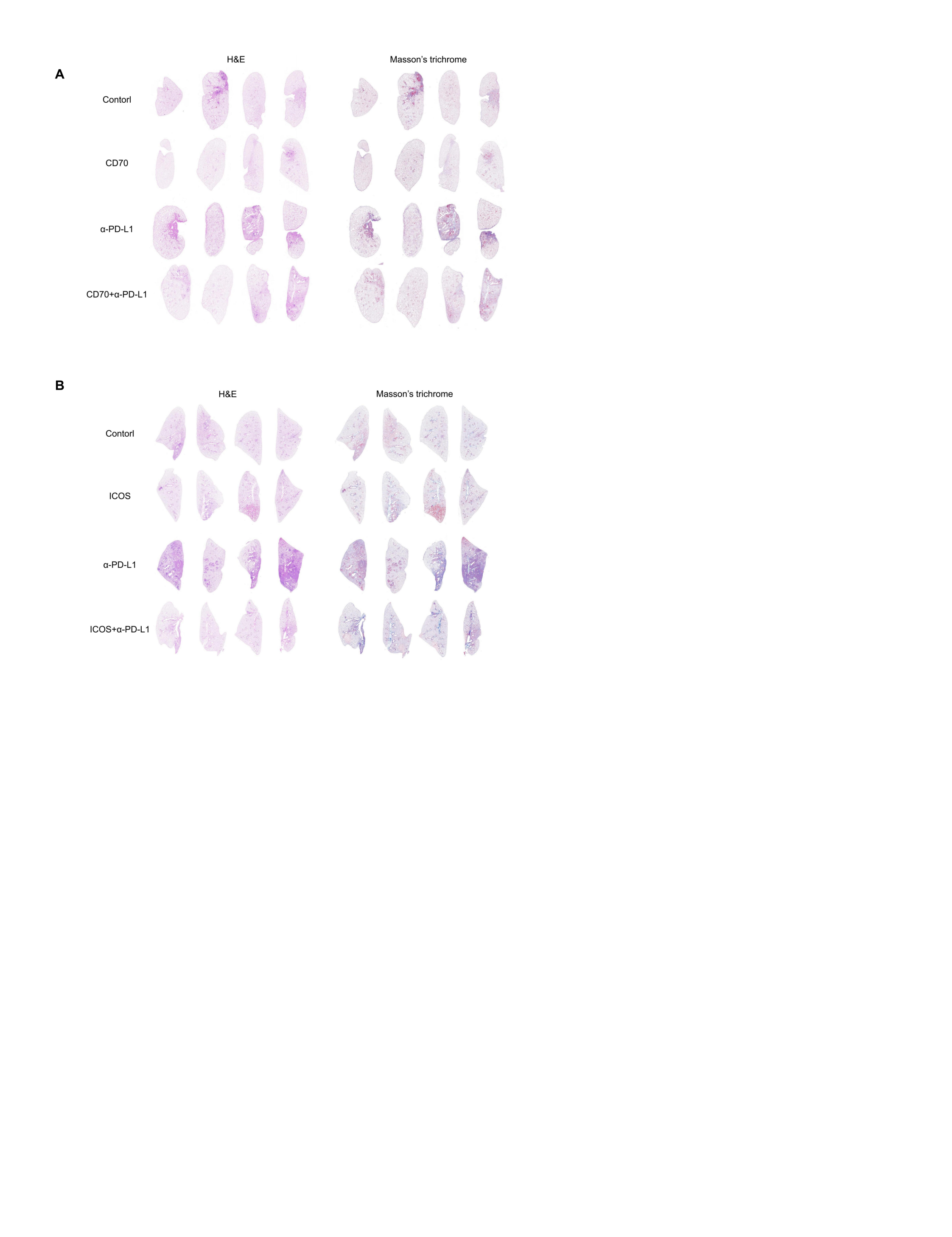


**Supplementary Figure 7**. **Blockade CD27-CD70 or ICOS-ICOSL interaction abrogates α-PD-L1 mediated lung fibrotic sequelae following influenza infection.** (**A** and **B**) WT C57BL/6 mice were infected with influenza PR8 and received either control IgG or the indicated antibodies from 21 to 37 d.p.i. Lung pathology were assessed at 60 d.p.i. Representative of three experiments (n = 4).


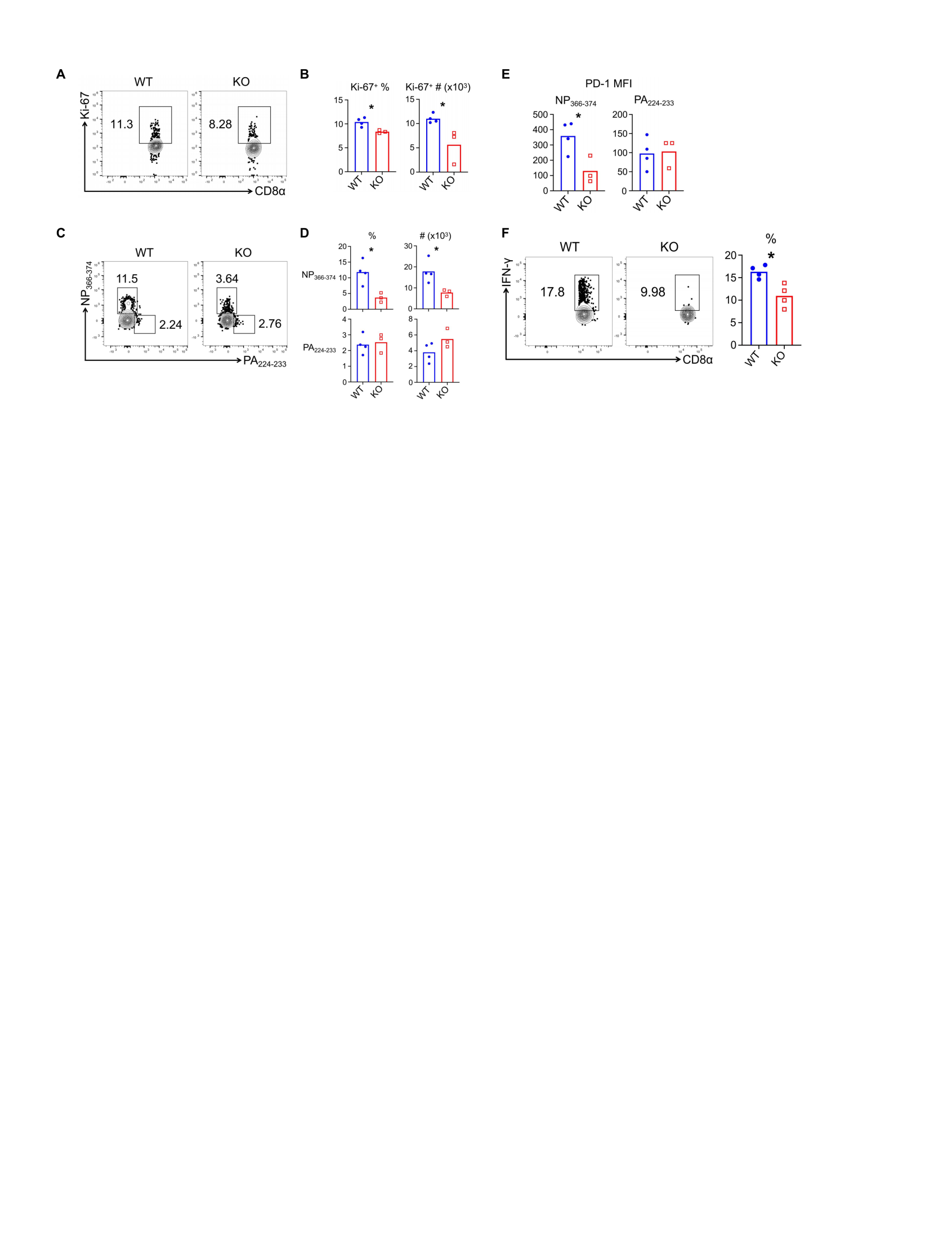


**Supplementary Figure 8. Lack of NR4A1 impairs the long-term maintenance of NP_366-374_ CD8^+^ T_RM_ cells.** (**A** to **F**) WT and NR4A1^-/-^ mice were infected with influenza PR8. (A and B) Representative plots, frequencies, and total cell numbers of Ki-67^+^ NP_366-374_ T_RM_ cells at 28 d.p.i. (C and D) Representative plots, frequencies, and total cell numbers of NP_366-374_ and PA_224-233_ T_RM_ cells at 42 d.p.i. (E) MFI of PD-1 on lung NP_366-374_ or PA_224-233_ T_RM_ cells at 42 d.p.i. (F) Representative plots and frequencies of IFN-γ^+^ NP_366-374_ T_RM_ cells at 28 d.p.i. Representative of three experiments (n = 4). Data are mean ± SD. *P < 0.05, unpaired two-tailed t test.


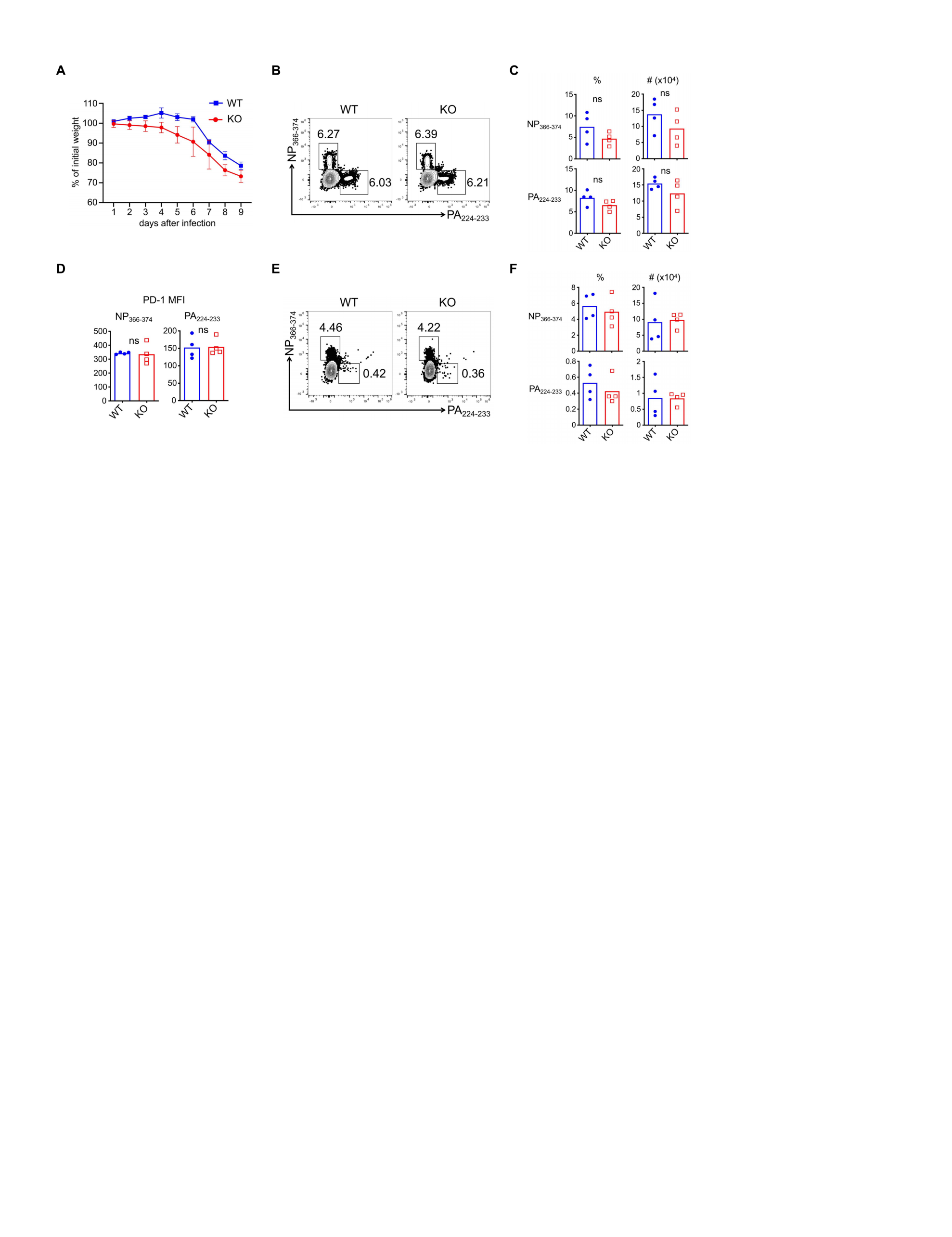


**Supplementary Figure 9**. **Lack of NR4A1 does not alter the effector CD8^+^ T cell response following influenza infection.** (**A** to **F**) WT and NR4A1^-/-^ mice were infected with influenza PR8. (A) Percentages of original body weight after primary infection were assessed daily. (B and C) Representative plots, frequencies, and total cell numbers of NP_366-374_ and PA_224-233_ CD8^+^ T cells in the lungs at 9 d.p.i. (D) MFI of PD-1 on lung NP_366-374_ or PA_224-233_ T_RM_ cells at 9 d.p.i. (E and F) Representative plots, frequencies, and total cell numbers of NP_366-374_ and PA_224-233_ CD8^+^ T cells in the spleen at 9 d.p.i. Representative of three experiments (n = 4). Data are mean ± SD. Unpaired two-tailed t test.


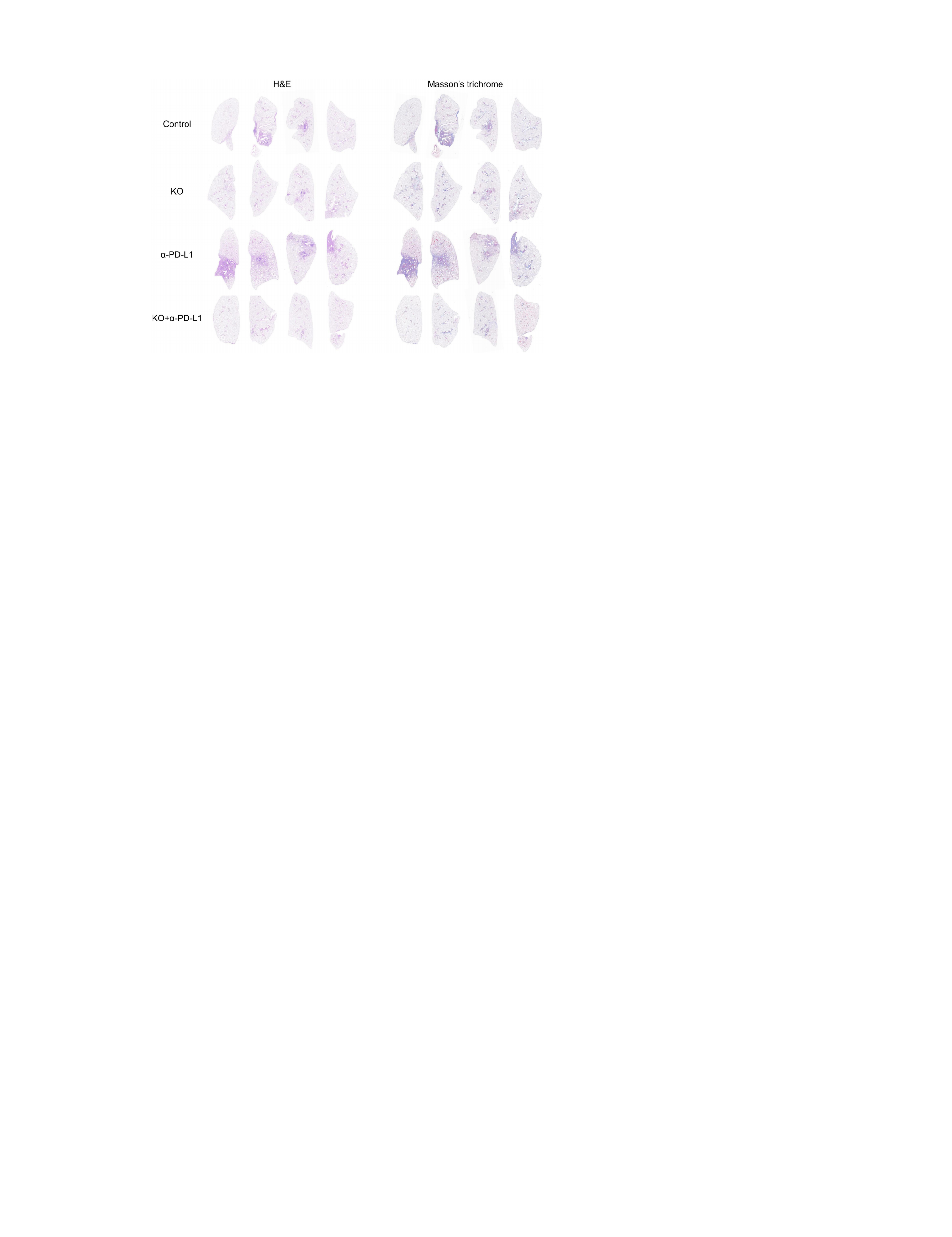


**Supplementary Figure 10. Lack of NR4A1 abrogates α-PD-L1 mediated lung fibrotic sequelae following influenza infection.** WT and NR4A1^-/-^ mice were infected with influenza PR8 and received either control IgG or α-PD-L1 from 21 to 37 d.p.i. Lung pathology were assessed at 60 d.p.i. Representative of three experiments (n = 4).


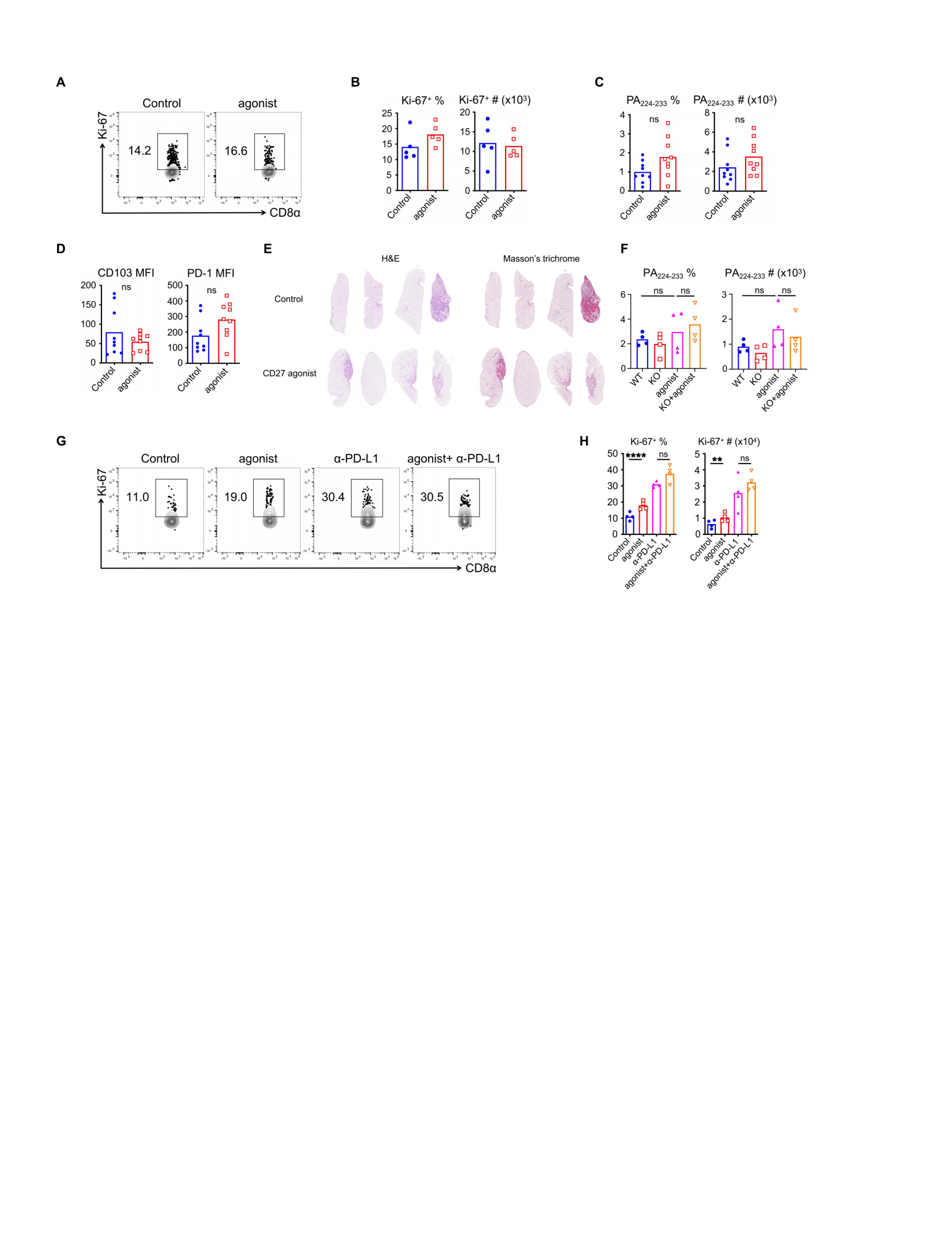


**Supplementary Figure 11**. **CD27 agonist provides host with better protection with reduced pathology.** (**A** to **E**) WT C57BL/6 mice were infected with influenza PR8 and received either control IgG or CD27 agonist from 21 to 25 d.p.i. or from 21 to 57 d.p.i. (A and B) Representative plots, frequencies, and total cell numbers of Ki-67^+^ NP_366-374_ T_RM_ cells at 29 d.p.i. (C) Frequencies and total cell numbers of PA_224-233_ T_RM_ cells at 60 d.p.i. (D) MFI of CD103 and PD-1 on lung NP_366-374_ T_RM_ cells at 60 d.p.i. (E) Lung pathology were assessed at 60 d.p.i. (**F**) WT and NR4A1^-/-^ mice were infected with influenza PR8 and received either control IgG or CD27 agonist from 21 to 37 d.p.i. Frequencies and total cell numbers of PA_224-233_ T_RM_ cells at 42 d.p.i. (**G** and **H**) WT C57BL/6 mice were infected with influenza PR8 and received control IgG, CD27 agonist, and/or α-PD-L1 from 21 to 34 d.p.i. Representative plots, frequencies, and total cell numbers of Ki-67^+^ NP_366-374_ T_RM_ cells at 35 d.p.i. Representative of three experiments (n = 4 to 9), data are mean ± SD; ns, not significant. **P < 0.01, ****P < 0.0001, unpaired two-tailed t test or one-way analysis of variance (ANOVA) with Tukey multiple comparison test.
